# Dynamic active-site generation of atomic iridium stabilized on nanoporous metal phosphides for water oxidation

**DOI:** 10.1038/s41467-020-16558-1

**Published:** 2020-06-01

**Authors:** Kang Jiang, Min Luo, Ming Peng, Yaqian Yu, Ying-Rui Lu, Ting-Shan Chan, Pan Liu, Frank M. F. de Groot, Yongwen Tan

**Affiliations:** 1 College of Materials Science and Engineering, Hunan University, Changsha, Hunan 410082 China; 2 Department of Physics, Shanghai Polytechnic University, Shanghai, 201209 China; 3 National Synchrotron Radiation Research Center, Hsinchu, 300 Taiwan; 4 State Key Laboratory of Metal Matrix Composites, School of Materials Science and Engineering, Shanghai Jiao Tong University, Shanghai, 200030 China; 5 Inorganic Chemistry & Catalysis, Debye Institute for Nanomaterials Science, Utrecht University, Universiteitsweg 99, 3584 CG Utrecht, The Netherlands

**Keywords:** Electrocatalysis, Electrocatalysis

## Abstract

Designing efficient single-atom catalysts (SACs) for oxygen evolution reaction (OER) is critical for water-splitting. However, the self-reconstruction of isolated active sites during OER not only influences the catalytic activity, but also limits the understanding of structure-property relationships. Here, we utilize a self-reconstruction strategy to prepare a SAC with isolated iridium anchored on oxyhydroxides, which exhibits high catalytic OER performance with low overpotential and small Tafel slope, superior to the IrO_2_. Operando X-ray absorption spectroscopy studies in combination with theory calculations indicate that the isolated iridium sites undergo a deprotonation process to form the multiple active sites during OER, promoting the O–O coupling. The isolated iridium sites are revealed to remain dispersed due to the support effect during OER. This work not only affords the rational design strategy of OER SACs at the atomic scale, but also provides the fundamental insights of the operando OER mechanism for highly active OER SACs.

## Abstract (web summary)

Direct observation of the atomic and electronic structure of a single-atom catalyst is essential. Here, the authors report an oxyhydroxide stabilized iridium catalyst with superior oxygen evolution catalytic activity and identify the isolated iridium sites which promote the H_2_O attack and O–O coupling.

## Introduction

Electrocatalytic water splitting for hydrogen and oxygen production provides an attractive path to obtain sustainable energy via the conversion and storage of intermittent solar and wind energies^1,2^. However, the bottleneck in improving water electrolysis is mainly caused by sluggish reaction kinetics at the anode, where water is oxidized and the oxygen evolution reaction (OER) occurs^2,3^. Currently, precious metal oxides, iridium (Ir), and ruthenium (Ru) oxides are the highly efficient OER electrocatalysts but suffer from relative scarcity and high-cost^4^. Though great efforts have been made to develop the earth-abundant transition-metal-based catalysts, including metal oxides or hydroxides^5–14^, borides^15,16^, phosphides^17–21^, sulfides^22,23^, and selenides^24,25^, as low-cost alternative for OER. However, their catalytic performance remains far from satisfactory. Thus, it is highly desirable to improve these OER systems, thus achieving high energy efficiency and cost-effectiveness in alkaline electrolysis.

Single-atom catalysts (SACs) offer a favorable pathway to maximize the catalytic activity while significantly reducing the amount of metals for chemical reactions^26–32^. The introduction of single atoms can largely improve the OER performance of support materials, such as carbon materials^27–30^, metals^31^, and metal oxide^32^. However, using metal phosphides as a support to anchor single-atom are still rare. They undergo surface self-reconstruction to form oxyhydroxides under OER conditions, providing abundant oxygen ligands for the anchoring of isolated atoms^18,19^. A fact that cannot be neglected is that most active sites on the surface of catalyst would undergo structural self-reconstruction due to the electro-derived structural oxidation processes^4,33^. Accordingly, it is reasonable to believe that the structural self-reconstruction of active sites would change the catalytic activity, especially for SACs. For instance, the aggregation of isolated atoms under extreme OER condition will lead to dramatic changes in the properties of catalysts. Therefore, a major challenge in the development of SACs is the difficulty to stabilize isolated atoms under drastic reaction conditions. Besides, stable SACs possessing the well-defined active centres offer an ideal model for exploring the origin of the activity under drastic OER conditions^34,35^. However, directly monitoring the dynamic behaviors of isolated active sites and identifying the synergy between different sites under extreme OER conditions are rarely available for SACs^28,30^, yet are pivotal for the development of efficient OER catalysts^35^. Consequently, it is highly desirable to develop a stable SACs with efficient OER performance and mechanistic insight into the single-atoms induced enhancement of OER activity.

In this work, we adopt a facile self-reconstruction strategy for single-atom Ir catalysts with controllable deposition of isolated Ir atoms on free-standing nanoporous (Ni_0.74_Fe_0.26_)_3_P (denoted as np-Ir/NiFeP). After subsequent electrochemical activation, the isolated Ir atoms turn into a stable state with higher valence and more oxygen ligands. The optimal catalyst (denoted as np-Ir/NiFeO) exhibits a high OER performance with low overpotential, high mass activity, and long stability, much superior to commercial IrO_2_ and most previously reported catalysts. Significantly, we identify, by employing operando X-ray absorption spectroscopy (XAS) and density functional theory (DFT) calculations, that the isolated Ir atoms undergo a variation from the single active site to multiple active sites through a deprotonation process during the OER process, which are responsible for the superior catalytic performance. Furthermore, the surface Ni(Fe) oxyhydroxides can form a shrinkage structure during OER conditions, further fixing the isolated Ir atoms, causing the excellent stability.

## Results

### Material synthesis and characterization

**Fig. 1:**
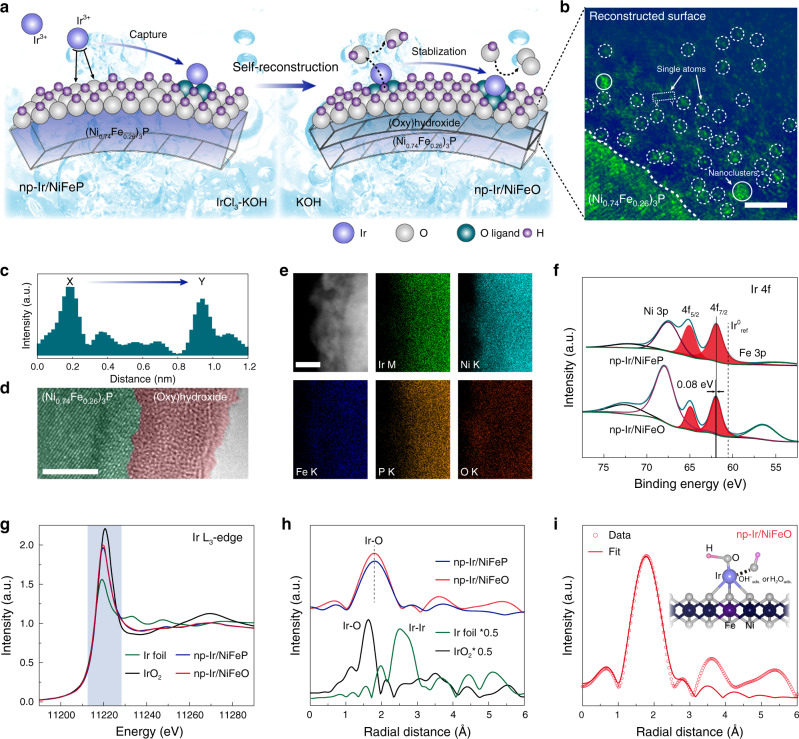
Structural characterization of np-Ir/NiFeO. **a** Schematic illustration of the preparation of np-Ir/NiFeO. **b** HAADF-STEM image of np-Ir/NiFeO, showing that Ir atoms appear as bright spots (highlighted by the white circles) and dispersed on the amorphous surface. **c** Intensity profiles along the lines X–Y in (**b**). **d** HRTEM image, showing the interface between (Ni_0.74_Fe_0.26_)_3_P and amorphous (oxy)hydroxides. **e** HAADF-STEM image and the corresponding EDX elemental mapping. **f** XPS spectra of np-Ir/NiFeP and np-Ir/NiFeO in Ir 4*f* region. **g** The normalized XANES at the Ir L_3_-edge of Ir foil, IrO_2_, np-Ir/NiFeP, and np-Ir/NiFeO. **h** Corresponding to FT-EXAFS spectra from (**g**). **i** First-shell (Ir–O) fitting of FT-EXAFS spectrum for np-Ir/NiFeO. Inset shows the structure of optimized np-Ir/NiFeO. Scale bar: **b** 2 nm, **d** 5 nm, **e** 5 nm.

The nanoporous (Ni_0.74_Fe_0.26_)_3_P (denoted as np-NiFeP) was synthesized using an electrochemically selective etching method (see “Method” section and Supplementary Figs. 1, 2)^36^. Then, Ir was atomically deposited on np-NiFeP by cyclic voltammetry (CV) using IrCl_3_–KOH as an electrolyte solution to produce the pre-catalyst (np-Ir/NiFeP) (Supplementary Figs. 3, 4). After electrochemical activation in basic solution, np-Ir/NiFeP underwent surface self-reconstruction to further stabilize isolated Ir atoms, forming the desired catalyst (np-Ir/NiFeO) (Fig. 1a, see “Method” section, and Supplementary Fig. 5). X-ray diffraction (XRD) patterns (Supplementary Fig. 6) show that both np-Ir/NiFeP and np-Ir/NiFeO have the dominant peaks identical to those of pristine np-NiFeP without characteristic peaks of Ir, which confirms the absence of Ir nanoparticles. While no peaks for Ni(Fe) (oxy)hydroxides were detected in the XRD patterns of np-Ir/NiFeO, revealing the formation of amorphous oxide layer. The Ni/Fe ratio approaches 1/3 in np-Ir/NiFeP (Supplementary Fig. 7), which correspond to the formation of stable Fe doped γ-NiOOH after electrochemical activation^19^. Representative high-angle annular dark-field scanning transmission electron microscopy (HAADF-STEM) images (Fig. 1b and Supplementary Fig. 8) at the reconstructed surface confirm that isolated Ir atoms (bright dots) uniform dispersed on the surface of amorphous Ni(Fe) (oxy)hydroxides. The single-atomic nature of Ir atoms and the amorphous nature of the reconstructed surface are further confirmed by line-scanning intensity profile (Fig. 1c) and high-resolution TEM (HRTEM, Fig. 1d and Supplementary Fig. 9). The STEM energy dispersive X-ray spectroscopy (EDX) elemental mapping shows the material is composed of Ir, Ni, Fe, P, and O elements (Supplementary Fig. 10), in which Ir is homogeneously distributed across the reconstructed surface (Fig. 1e).

The chemical composition and oxidation states of catalysts were analyzed by X-ray photoelectron spectroscopy (XPS). Figure 1f shows deconvolution of the Ir 4f core level spectra, which exhibits a doublet (Ir 4*f*_7/2_ and 4*f*_5/2_) at 62.0 eV and 65.0 eV for np-Ir/NiFeP, indicating Ir atoms carry positive-charge in relation to metallic Ir due to the electronic interaction between Ir atoms and support^37^. After surface self-reconstruction, the doublet of np-Ir/NiFeO exhibits a positive-shift compared with that of Ir/NiFeP, underscoring the higher Ir valence state of np-Ir/NiFeO. The core-level Ni 2*p* XPS spectrum of np-NiFeP displays three peaks with binding energies of 852.8, 856.0, and 861.6 eV assigned to Ni_3_P, Ni oxide or hydroxide, and relevant satellite peak, respectively (Supplementary Fig. 11a)^20,38^. Meanwhile, two peaks with binding energies located at 706.5 and 712.8 eV in core-level Fe 2*p* XPS spectrum of np-NiFeP are ascribed to Fe_3_P and Fe oxide (Supplementary Fig. 11b)^18^. Compared with np-NiFeP, the Ni 2*p* and Fe 2*p* XPS spectra of np-Ir/NiFeP show a redistribution of more Ni (Fe) oxides or hydroxides from Ni_3_P (Fe_3_P) and phosphate due to the presence of alkaline solution and the reduction trend of the cathodic voltage (Supplementary Fig. 11c)^24^. During the atomic deposition process, Ir species are easily trapped by oxygen ligands and stabilized thereupon, producing np-Ir/NiFeP^39^. After electrochemical activation, the Ni 2*p* and Fe 2*p* XPS spectra confirm that a mixture of Ni(Fe) (oxy)hydroxides evolves at the surface of the np-Ir/NiFeO, which changes the coordination environment of isolated Ir atoms^18^.

The X-ray absorption near-edge structure (XANES) spectroscopy and extended X-ray absorption fine structure (EXAFS) spectroscopy were measured to further probe the electronic and coordination structures of the catalysts^40^. The white line intensity of np-Ir/NiFeP in Ir L_3_-edge XANES spectrum is situated between those of Ir foil and IrO_2_, indicating a cationic environment (Fig. 1g)^37^. After electrochemical activation, the white line intensity of np-Ir/NiFeO increased, indicating the further oxidation of the Ir species. As shown in the Fourier transform of EXAFS (FT-EXAFS) spectra (Fig. 1h), np-Ir/NiFeP exhibits a prominent peak at 1.78 Å corresponding to Ir–O scattering feature^37,41^. No typical peaks for Ir-Ir contribution are observed, revealing the isolated dispersion of Ir atoms. Compared with np-Ir/NiFeP, the intensity of Ir–O shell increases in the FT-EXAFS spectrum of np-Ir/NiFeO, indicating that isolated Ir atoms turn into a more stable state with higher valence and more oxygen ligands after surface self-reconstruction (Supplementary Fig. 11d)^39^. Moreover, FT-EXAFS fitting of np-Ir/NiFeO based the structure model in DFT (Fig. 1i and Supplementary Table 3) demonstrates that each Ir atom is coordinated by four O atoms from oxygen ligands and two O atoms from absorbed hydroxyl or water group^30^, further demonstrating the single-atomic nature of Ir. The Ni K-edge XANES spectra for np-NiFeP, np-Ir/NiFeP, and np-Ir/NiFeO are shown in Supplementary Fig. 12a. The white line intensity of np-Ir/NiFeP is slightly higher than that of np-NiFeP, implying the partial oxidation of the Ni species and the formation of an oxygen-enriched surface which provides the anchoring ligand for the Ir species^8,42^. After the activation process, the absorption-edge of np-Ir/NiFeO shifts to higher energy, in combination with an increase in the white line intensity due to the formation of a Ni (oxy)hydroxide layer^9^. The corresponding FT-EXAFS spectrum of np-Ir/NiFeP shows a decrease of the peak at ~1.95 Å in comparison with that of np-NiFeP (Supplementary Fig. 12b), which can be attributed to the break of Ni–P bonds during the generating of Ni oxides or hydroxides. Furthermore, compared with np-Ir/NiFeP, the prominent peak of np-Ir/NiFeO shows a low-*R* shift by 0.06 Å, along with the rise and broadening of the peak, which is ascribed to a contribution of Ni–O bond from Ni (oxy)hydroxides that partial overlapped with the Ni–P bond^21^. In addition, the peak located at 2.81 Å (denoted as peak I) could be viewed as a signal of Ni (oxy)hydroxides. Thus, the higher peak I in np-Ir/NiFeO further implies the formation of Ni (oxy)hydroxides^7,10^. In the case of Fe, XANES and FT-EXAFS results display similar phenomena to Ni (Supplementary Fig. 12c, d), which confirms that the surface Fe species have the same function and behavior as the surface Ni species.

### Electrochemical oxygen evolution performance

**Fig. 2:**
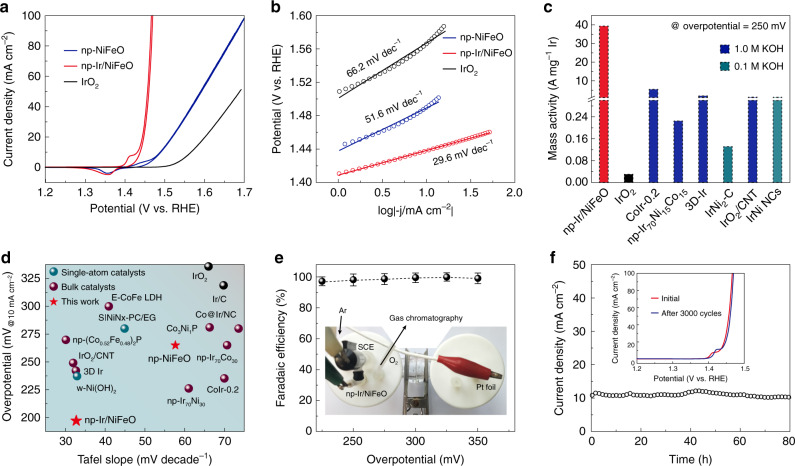
Electrochemical OER performance. **a** OER polarization curves of np-NiFeO, np-Ir/NiFeO, and IrO_2_. **b** Corresponding to Tafel plots of the presented data in (**a**). **c** The mass activities of np-Ir/NiFeO, IrO_2_, and state-of-the-art OER catalysts. **d** Comparison of Tafel slope and overpotential required to achieve 10 mA cm^−2^, with references all measured in alkaline medium. **e** Gas chromatography analyses for np-Ir/NiFeO. Inset shows the detail of Faradaic efficiency measurement. Error bars represent the standard deviation from multiple measurements. **f** The current density versus time (i–t) curves of np-Ir/NiFeO recorded for 80 h at 1.43 mV versus RHE. Inset shows the CV curves of np-Ir/NiFeO before and after the acceleration durability test for 3000 cycles.

The OER activities of np-NiFeO and np-Ir/NiFeO were evaluated in a three-electrode set-up, along with commercial IrO_2_ as reference. Figure 2a shows the linear sweep voltammetry (LSV) of those catalysts in O_2_-saturated KOH solution. The np-Ir/NiFeO shows the highest performance among all samples, requiring overpotential of only 170 and 197 mV to reach a current density of 1 (defined as onset overpotential) and 10 mA cm^−2^, significantly better than those of np-NiFeO and commercial IrO_2_ (Supplementary Fig. 13). Strikingly, np-Ir/NiFeO electrode can achieve a high current density up to 300 mA cm^−2^ at 1.48 V versus reversible hydrogen electrode (RHE) because of enhanced transfer of mass (reactants and oxygen bubbles) (Supplementary Fig. 14), a large specific surface area, and efficient gas pumping of bicontinuous nanoporous structure (Supplementary Fig. 15). A small Tafel slope of 29.6 mV per decade (mV dec^−1^) is measured for np-Ir/NiFeO (Fig. 2b), which is much smaller than np-NiFeO (51.6 mV dec^−1^) and IrO_2_ (66.2 mV dec^−1^). The lower Tafel slope and charge transfer resistance (Supplementary Fig. 16) of np-Ir/NiFeO in relation to np-NiFeO demonstrate that np-Ir/NiFeO is endowed with the favorable fast oxygen evolution kinetics by the introduction of isolated Ir atoms. Additionally, normalized to the Ir loading (0.1 wt%, Supplementary Fig. 17), the mass activity of np-Ir/NiFeO for OER at an overpotential of 250 mV is 39.3 A mg^−1^, which is 131 times greater than that of the IrO_2_ catalyst (0.30 A mg^−1^) (Supplementary Note 1). This result indicates that isolated Ir atoms anchored on np-NiFeO support can maximize the catalytic activity, allowing significant cost reduction of the OER catalyst. Compared with previously reported OER catalysts, np-Ir/NiFeO shows the higher catalytic performance in terms of mass activity, overpotential at 10 mA cm^−2^, and Tafel slope in the alkaline solution (Fig. 2c, d and Supplementary Tables 1, 2)^5,17,22,27,37,43^.

To elucidate the origin of the huge improvement of the OER activity, a CV method was utilized to measure the electrochemical double-layer capacitances (C_dl_) of corresponding catalysts for evaluating the electrochemically effective surface areas (ECSA)^18^. Our results show a considerably larger C_dl_ of np-Ir/NiFeO (21.4 mF cm^−2^) compared with np-NiFeO (14.9 mF cm^−2^), manifesting that the new generated Ir sites act as the accessible active sites on np-Ir/NiFeO (Supplementary Fig. 18). The ECSA-normalized CV curves are employed to emphasize the intrinsic activity (Supplementary Note 2 and Supplementary Fig. 19). It is distinct that the ECSA-normalized current density of np-Ir/NiFeO is larger than that of np-NiFeO, indicating that the higher OER activity of np-Ir/NiFeO results from not only the increased ECSA but also the enhanced intrinsic activity. Besides, gas chromatography analyses show that np-Ir/NiFeO exhibits the O_2_ Faraday efficiency of near 100% under different applied potentials (Fig. 2e). During a chronoamperometry measurement at 1.43 V on np-Ir/NiFeO, the anodic current shows no evidence of degradation over 80 h (Fig. 2f). An accelerated CV cycling test was performed to verify this catalytic robustness with negligible shift of polarization curves after 3000 cycles (Fig. 2f inset). Further insights into the morphology for post-OER np-Ir/NiFeO by scanning electron microscopy (SEM) confirm the retaining of nanoporous structure (Supplementary Fig. 20). XAS measurement of np-Ir/NiFeO after long-time stability test displays some predictable changes (Supplementary Fig. 21), namely, all the metallic elements had relatively oxidized after long-time operation, but keeping the atomic dispersion of Ir atoms.

### OER enhancement mechanism

**Fig. 3:**
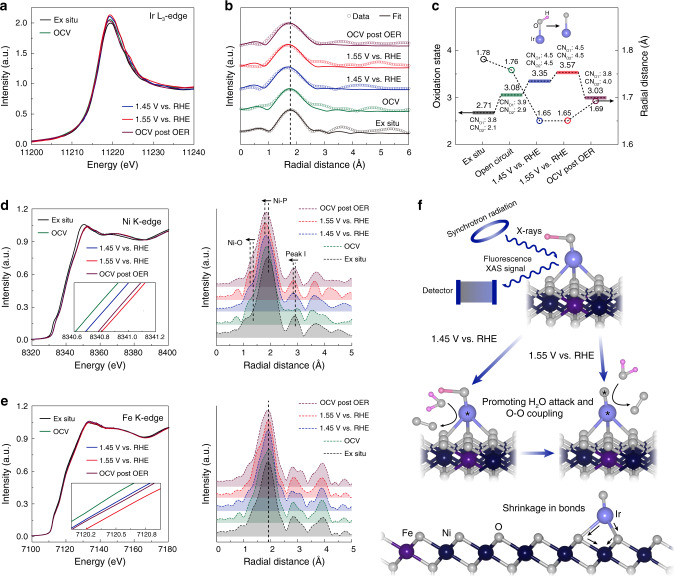
Operando XAS characterizations. **a** Operando XANES spectra of np-Ir/NiFeO recorded at Ir L_3_-edge under different applied voltages from OCV to 1.55 V versus RHE in 1.0 M KOH. **b** Corresponding first-shell (Ir–O) fitting of FT-EXAFS spectra for np-Ir/NiFeO. **c** The fitted oxidation states from the white line intensity analysis, the variation of Ir–O bond, and the FT-EXAFS curve-fitting analysis. **d** Operando XANES spectra of np-Ir/NiFeO recorded at Ni K-edge under different applied voltages from OCV to 1.55 V versus RHE in 1.0 M KOH, as well as the corresponding FT-EXAFS spectra. **e** Operando XANES spectra of np-Ir/NiFeO recorded at Fe K-edge under different applied voltages from OCV to 1.55 V versus RHE in 1.0 M KOH, as well as the corresponding FT-EXAFS spectra. **f** Schematic illustration of the OER mechanism determined by the operando XAS analysis of np-Ir/NiFeO.

To elucidate the origin of the outstanding OER catalytic performance and the structural advantages of np-Ir/NiFeO, the dynamic changes in oxidation state and local coordination environment at OER-relevant potentials was probed by operando XAS analysis^40^. Figure 3a, b display the normalized operando Ir L_3_-edge XANES spectra and the corresponding FT-EXAFS spectra. Meanwhile, the fitted oxidation states from the white line intensity analysis, the variation of Ir–O bond, and the FT-EXAFS curve-fitting analysis are shown in Fig. 3c, Supplementary Figs. 22, 23, and Supplementary Table 3. The analysis of Ir coordination shell was carried out by taking two backscattering paths, including Ir–O1 (the O ligands and intermediate species) and Ir–O2 (the chemisorption of OH^−^ and H_2_O). Under the open-circuit voltage (denoted as OCV), np-Ir/NiFeO shows an increase of white line intensity in XANES spectrum compared with the ex-situ condition, resulting from the chemisorption of OH^− ^and H_2_O^30,44^. This is also evidenced by the corresponding FT-EXAFS analysis, which shows the increase in the coordination number of Ir–O2 (denoted as CN_O2_). During electrochemical OER (1.45 V versus RHE), a further increase of the white line intensity occurs in relation to the case under OCV, implying the formation of Ir intermediate species (such as OOH*) and the more chemisorption of OH^− ^and H_2_O^44,45^. This variation of atomic coordination environment also reflects by the increase in the coordination number of Ir–O1 (denoted as CN_O1_) and the CN_O2_ in the corresponding FT-EXAFS analysis. However, as the further increase of anodic potential (1.55 V versus RHE), np-Ir/NiFeO only displays the increase of white line intensity in XANES spectrum without the change of FT-EXAFS spectrum in comparison with that under 1.45 V versus RHE. This result manifests that the increase of the valance state of Ir under higher anodic potential was not result from the further chemisorption or the formation of intermediate species. Therefore, we propose a deprotonation process (Ir–OH to Ir–O*) which occurs on Ir sites under higher applied potential. The deprotonation step will cause the oxidation of center atom while not change the atomic coordination environment^4^. The active oxygen species created during the deprotonation step play a critical role in efficient OER process, which serves as a synergistic site to promote the H_2_O attack and O–O coupling^6,46,47^, resulting the faster OER kinetics under higher applied potential. When the potential restores back to OCV, the valance state of Ir for np-Ir/NiFeO can recover to its initial state, implying the restorability in the oxidation state of Ir. Additionally, it is clear that Ir exists as isolated atoms during the OER process without agglomeration, which is attributed to the shrinkage of Ir–O bonds under realistic reaction conditions (Fig. 3b and Supplementary Table 3).

In the case of Ni, the absorption-edge of np-Ir/NiFeO in XANES spectrum (Fig. 3d) displays an obvious positive shift under OCV condition in relation to the ex situ condition, manifesting the oxidation of the Ni species^48^. Correspondingly, the main peak in the FT-EXAFS spectrum shows a low-*R* shift, meaning the change of local atomic structure. During electrochemical OER (1.45 and 1.55 V versus RHE), the further oxidation behavior of Ni species is observed by the positive shift of the absorption-edge, indicating the transformation from hydroxides to oxyhydroxides in np-Ir/NiFeO. Correspondingly, the FT-EXAFS spectrum shows the negative-shift of all peaks (Ni–O, Ni–P, and peak I), especially for Ni–O peak (splitting from main peak)^7^, indicating the shrinkage of bonds in np-Ir/NiFeO. This shrinkage in Ni–O bonds could further fix the isolated Ir atoms on the surface of np-Ir/NiFeO, thus avoiding possible agglomeration during OER^39^. In particular, when the potential restores back to OCV, the valance state of Ni cannot recover to its initial state, suggesting the irreversibility in the oxidation state of Ni during the OER process. In the case of Fe, similar changes in XANES spectra (Fig. 3e) suggest the similar behavior of Ni and Fe. Unlike Ni, the main peak in Fe FT-EXAFS spectra exhibits constant radial distance. This could be resulted from the less content of Fe compared with Ni on the surface of np-Ir/NiFeO, weakening the variation during OER process. Besides, further insights into the regulation effect of Ir atoms incorporation by controlled operando XAS measurement of np-Ir/NiFeO and np-NiFeO confirm that more transformation from Ni(Fe) oxides or hydroxides to Ni(Fe) oxyhydroxides occurs on the surface of np-Ir/NiFeO than np-NiFeO, as evidenced by the greater shifts of the absorption-edge of np-Ir/NiFeO compared with that of np-NiFeO under OER conditions (Supplementary Fig. 24)^9^. Considering the existence of strong metal-support interactions in SACs^34,35^, it is reasonable to believe that the charge transfer from the Ni(Fe) oxides or hydroxides to Ir atoms at the interfacial sites under realistic OER conditions is responsible for the facilitating of oxidation of Ni(Fe) oxides or hydroxides to Ni(Fe) oxyhydroxides, thus enabling an earlier onset of current for the OER^49,50^.

Based on the above operando XAS results, we propose a straightforward mechanism for the high OER activity and stability based on np-Ir/NiFeO (Fig. 3f). During electrochemical OER, the OH ligands of Ir atoms undergo a deprotonation process and turn into new stable oxygen sites with the increase of applied anodic potential. Multiple active centers consisting of Ir sites and active oxygen species accelerate OER process under extremely conditions. Meanwhile, the shrinkage of the Ni(Fe)–O bonds leads to a more stable surface structure, thus fixing the isolated Ir atoms and preventing agglomeration. In addition, it can be seen that the incorporation of Ir atoms promotes the formation of Ni(Fe) oxyhydroxides, further accelerating the reactions.

### Theoretical investigations on OER activity

**Fig. 4:**
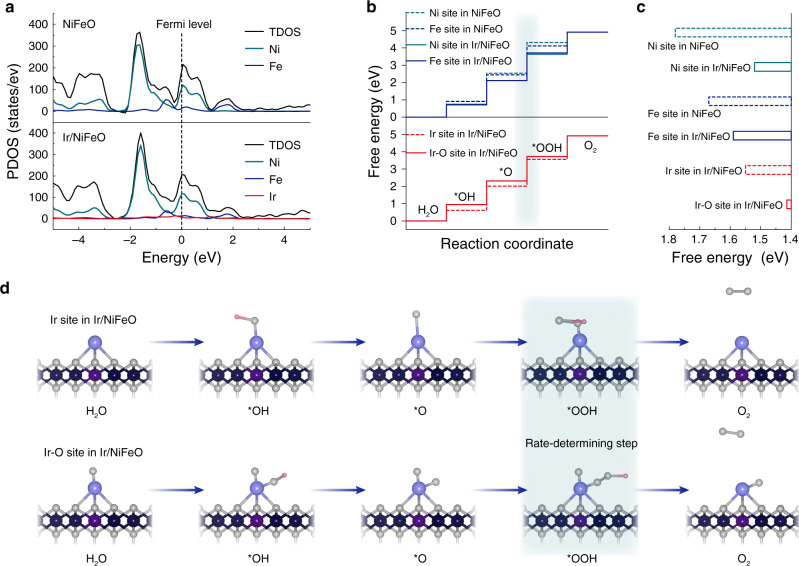
DFT calculations. **a** Calculated PDOS of NiFeO and Ir/NiFeO. **b** Calculated free energy diagram of the OER. The blue box step is the rate-determining step. **c** The Gibbs free energy of the rate-determining step for different sites. **d** Schematic of the whole OER mechanism on Ir/NiFeO in the alkaline electrolyte.

To further understand the OER mechanism of np-Ir/NiFeO, theoretical investigations were performed based on DFT calculations^27,51–57^. The projected density of states (PDOS) results suggest that the introduction of Ir species endows new hybridized electronic states in Ir/NiFeO, resulting the widening of the total density of states (TDOS) near the Fermi level (Fig. 4a). This change is main attributed to the Ni and Fe 3*d* orbitals^29^, indicating that the *d*-electron domination of surface Ni and Fe atoms were optimize by the introduction of single-atom Ir, which leads to the increase of the effective charge concentration on the surface of Ir/NiFeO. Furthermore, the elementary reaction steps for the OER process in alkaline environments are demonstrated in Fig. 4b, Supplementary Figs. 25–29, Supplementary Note 3, and Supplementary Table 4. For pure NiFeO, Tafel slope shows that the O–O coupling (the transition from O* to OOH*) is the rate-determining step under alkaline conditions^7^. Thus, the large Gibbs free energy of the rate-determining step for Ni sites and Fe sites can slow down and even block the O_2_ evolution, resulting in an extremely sluggish OER kinetics (Fig. 4c). After the introduction of the Ir species, the Gibbs free energy of the rate-determining step for Ni sites and Fe sites dramatically decreased, suggesting a more favorable OER kinetics for Ni and Fe sites. Significantly, Ir sites in Ir/NiFeO have the low Gibbs free energy of the rate-determining step than that of Ir sites on (001) face of Ir crystal, indicating that isolated Ir sites on Ir–NiFeO are efficient active sites to catalyze OER (Supplementary Fig. 29). Afterward, we move onto the Ir–O moiety generating under realistic reaction conditions (Fig. 4d). It is found that the Gibbs free energy of the rate-determining step for the Ir–O moiety is substantially lower than that of other sites, which suggests the facilitation of nucleophilic H_2_O attack and O–O coupling during OER process, in coincidence with the aforementioned operando XAS results. Consequently, the activation of Ni(Fe) oxyhydroxides and the formation of Ir–O moiety are responsible for the excellent OER activity of Ir/NiFeO.

## Discussion

In summary, we developed a novel single-atom Ir incorporated into Ni(Fe) oxyhydroxides catalyst for OER, by utilizing the surface reconstruction of nanoporous Ni (Fe) phosphides. The as-prepared np-Ir/NiFeO catalyst exhibited an outstanding OER performance in alkaline aqueous solutions, showing an OER overpotential of 197 mV versus RHE to achieve a current density of 10 mA cm^−2^, an ultralow Tafel slope of 29.6 mV dec^−1^, a high mass activity of 39.3 A mg^−1^ (131 times higher than that of the commercial IrO_2_), and excellent cycling stability. These impressive OER performances are comparable to, or even better than the values of state-of-the-art catalyst materials, including the commercial IrO_2_ catalyst. Through operando XAS analysis and DFT calculations, we find that the isolated Ir atoms vary from single active site to multiple actives through a deprotonation processes under realistic reaction conditions, leading to the excellent OER performance. The superior activity could be attributed to the Ir–O moiety, manifested in favorable formation of the OOH* intermediate in the OER process. Meanwhile, the strong electron interaction between isolated Ir atoms and (oxy)hydroxides results in a more stable surface structure during OER process, confirming the excellent stability of isolated Ir atoms. Besides, the incorporation of Ir atoms could promote the formation of Ni(Fe) oxyhydroxides and arouse the intrinsic activity of Ni(Fe) oxyhydroxides, further accelerating the reactions. Thus, this work presented herein highlight the importance of using operando XAS to identify the fine structure of the reconstruction-derived components and deepen the understanding of the dynamic structure of single-atomic electrocatalysts in promoting OER activity.

## Methods

### Materials synthesis

The np-NiFeP was synthesized through electrochemically selective etching. Then, repeated CV scans was conducted to perform controllable deposition. Specifically, the IrCl_3_ powder (1.0 mg) was added to 500 mL of KOH solution (1.0 M) and stirred for 2 h to obtain the homogeneous mixture as an electrolyte. The deposition process was carried out by 750 CV cycles with a voltage range from 0.075 to −0.475 V versus RHE at a scan rate of 50 mV s^−1^. Finally, the self-reconstruction process was conducted in fresh KOH (1.0 M) by 50 CV cycles with a voltage range from 1.0 to 1.6 V versus RHE at a scan rate of 50 mV s^−1^.

### Characterization

XRD patterns were acquired by using a Bruker D8 advance (Cu Kα radiation, λ = 1.5418 Å). SEM measurements were performed on Zeiss Sigma HD (Oxford EDS). HRTEM images, HAADF-STEM images, and EDX mappings were obtained by using a JEM-ARM 200F. XPS date were achieved on Thermo Fisher Scientific ESCALAB250Xi spectrometer (Al Kα monochromatic). The specific surface area was collected based on the Brunauer-Emmet-Teller (BET) method using Micromeritics ASAP 2020. Inductively coupled plasma optical emission spectrometry (ICP-OES) was performed on Agilent 730.

### Electrochemical testing

All electrochemical OER activity and durability were measured using an Ivium CompactStat.h electrochemical workstation with np-Ir/NiFeO as the working electrode, a graphite sheet as the counter electrode, and a saturated calomel electrode (SCE) as the reference electrode in O_2_-saturated 1.0 M KOH. CV results were obtained in the range of 1.0 to 1.6 V versus RHE at a scan rate of 2 mV s^−1^. All potentials were converted to the RHE scale and iR-corrected by the resistance of the electrolyte. To minimize the masking effect of redox couple, we derived the Tafel plots and compared the OER activities using the cathodic sweep. The OER durability were measured by repeating the potential scan from 1.2 to 1.6 V versus RHE at a sweep rate of 50 mV s^−1^ with 3000 CV cycles. Chronoamperometric characterization was performed at a potential of 1.43 V versus RHE for 80 h.

### Operando XAS measurements

The Ir L_3_-edge, Ni K-edge, and Fe K-edge XAS spectra were measured at the beamline BL01C1 of National Synchrotron Radiation Research Center (NSRRC, Taiwan). Operando XAS measurements were performed using an Ivium CompactStat.h electrochemical workstation and a home-built cell equipped with a carbon rod counter electrode and a SCE reference electrode. The working electrode was prepared by dropping the catalyst ink on the carbon cloth. The fresh KOH electrolyte was bubbled with pure oxygen for 1 h. For installation of operando XAS setup, the side of working electrode covered with Kapton film was faced to the incident X-rays. Simultaneously, the other side of working electrode was contacted with KOH electrolyte^40^. The XAS spectra were measured in the fluorescence mode at room temperature. During the operando experiments, the OER-relevant potentials of OCV, 1.45, and 1.55 V versus RHE were applied to this system. Acquired XAS data were processed with the ATHENA program.

### DFT calculations

In this paper, the DFT + U calculations were performed with the Vienna ab initio simulation package (VASP)^54^. The exchange-correlation potential was treated by using the generalized gradient approximation (GGA) of Perdew–Burke–Ernzerhof (PBE) functional^55,56^. In the calculations, the value of *U* is 3.8 for Ni, 4.3 for Fe, and 0 for Ir (no *U* correction), which was selected according to the previous studies^57^. The (001)-terminated surface was selected in the 5 × 5 × 2 γ-NiOOH and a vacuum slab of 15 Å was added along the surface. The cutoff energy was set as 450 eV. During structural optimization and the self-consistent calculations, the Brillouin zone (BZ) was sampled by a Monkhorst-Pack k-mesh of 5 × 5 × 1. The unit cell was optimized until the force and total energy were set to be 0.01 eV/Å and 10^−5^ eV, respectively.

## Supplementary information

Supplementary Information

Peer Review

## Data Availability

The data that support the findings of this study are available from the corresponding authors upon reasonable request.
