# Supplementary material for: Dynamic active-site generation of atomic iridium stabilized on nanoporous metal phosphides for water oxidation
Source: Nat Commun. 2020 Jun 1;11:2701. doi: 10.1038/s41467-020-16558-1 (PMC7264278; doi:10.1038/s41467-020-16558-1)
Supplement: Supplementary file 1 — Supplementary Information [file 41467_2020_16558_MOESM1_ESM.docx]

**Supplementary Information**

**Dynamic active-site generation of atomic iridium stabilized on nanoporous metal phosphides for water oxidation**

Jiang et al.


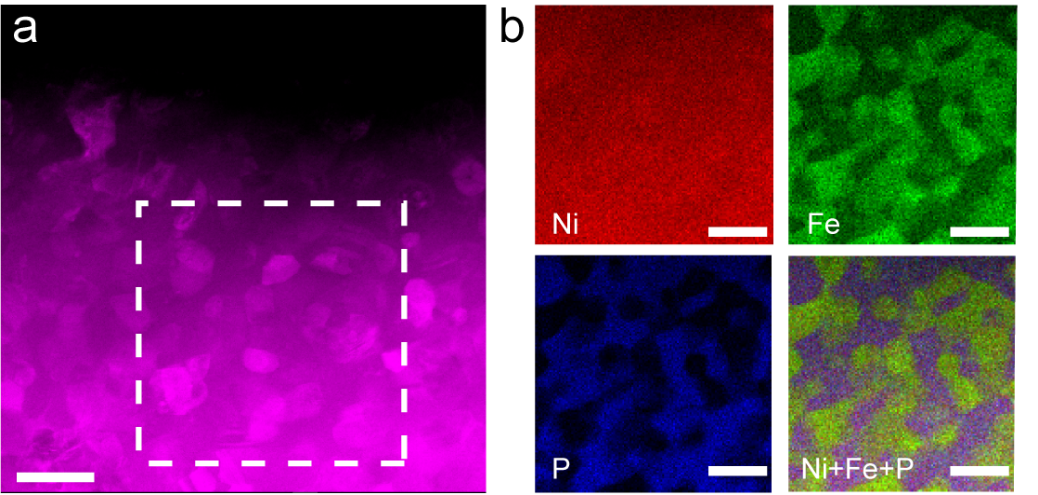


**Supplementary Figure 1.** **TEM characterizations of Ni_55_Fe_35_P_10_ ribbons**

(**a**) HAADF-STEM image of the Ni_55_Fe_35_P_10_ ribbons. The marked box is the region selected for EDS chemical analysis. (**b**) STEM-EDS element mappings of Ni_55_Fe_35_P_10_ ribbons taken from the marked region in (a), showing phase separation of (Ni_x_Fe_1-x_)_3_P and NiFe. Scale bar: (**a**, **b**) 50 nm.


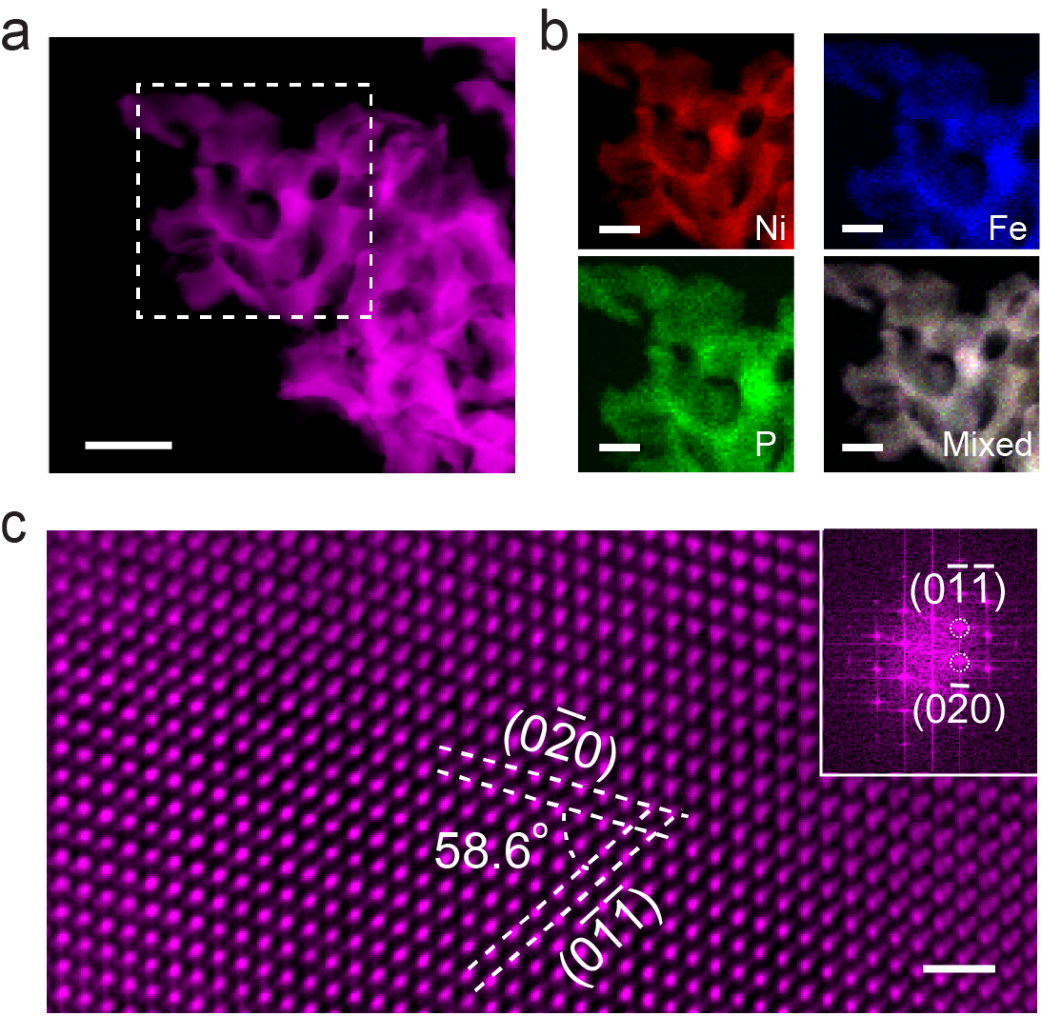


**Supplementary Figure 2.** **TEM characterizations of (Ni_0.74_Fe_0.26_)_3_P ribbons**

(**a**) HAADF-STEM image. The marked box is the region selected for EDS chemical analysis. (**b**) STEM-EDS element mappings of (Ni_0.74_Fe_0.26_)_3_P ribbons taken from the marked region in (a). (**c**) Fast Fourier transformation (FFT) filtered HAADF-STEM image. Inset: corresponding FFT pattern. Scale bar: (**a**) 100 nm, (**b**) 50 nm, (**c**) 1 nm.


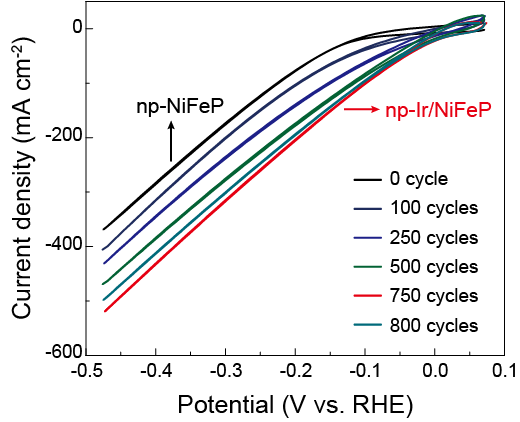


**Supplementary Figure 3.** **Controllable Ir deposition**

CVs of np-NiFeP scanned in Ar-saturated IrCl_3_-KOH solution at a scan rate of 50 mV s^-1^ (without corrected for *iR* losses).


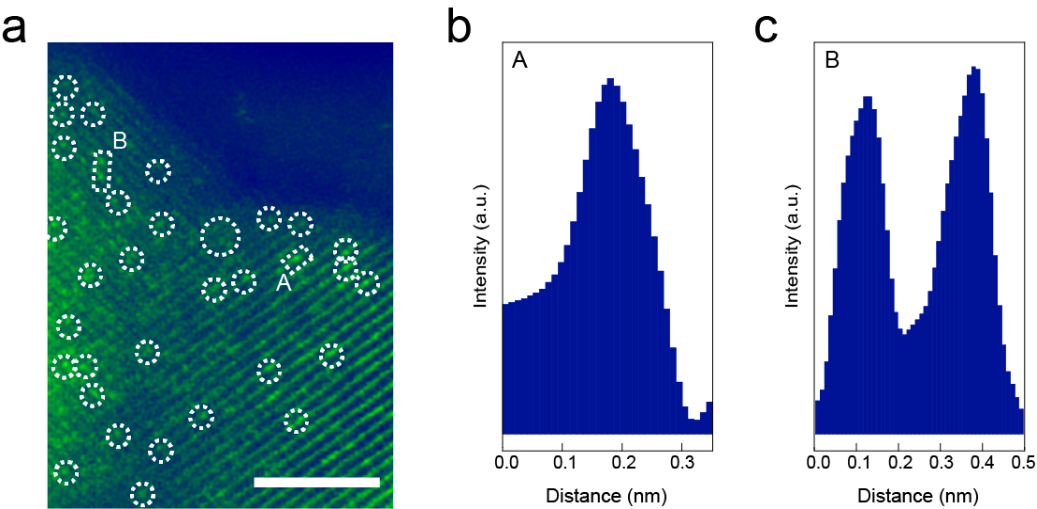


**Supplementary Figure 4.** **HAADF-STEM characterizations of np-Ir/NiFeP**

(**a**) HAADF-STEM image, showing the surface of nanoporous (Ni_0.74_Fe_0.26_)_3_P with isolated Ir atoms doping. The isolated Ir atoms were highlighted by the white circles. (**b**, **c**) The line-scanning intensity profile obtained from the area highlighted with white rectangles in (a). Scale bar: (**a**) 2 nm.


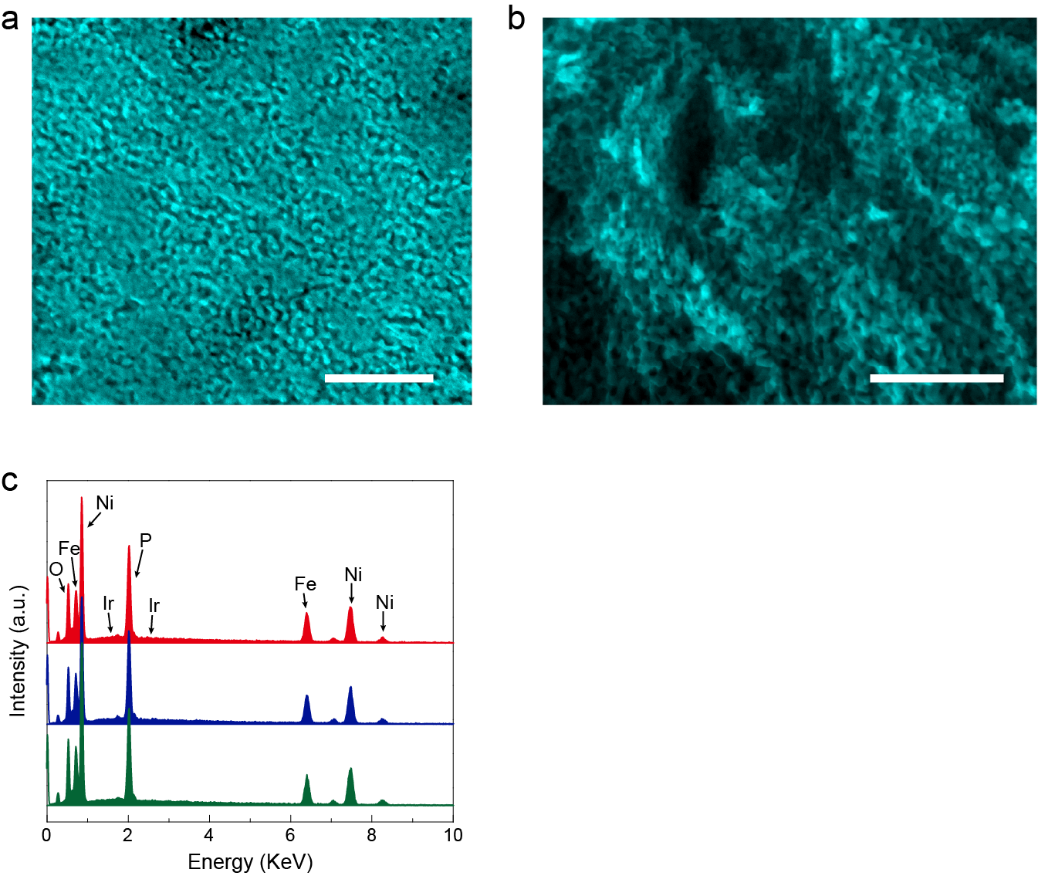


**Supplementary Figure 5.** **SEM characterizations of np-Ir/NiFeO**

(**a**) SEM image and (**b**) cross-section SEM image of np-Ir/NiFeO. (**c**) Corresponding EDS spectra. Scale bar: (**a**, **b**) 500 nm.


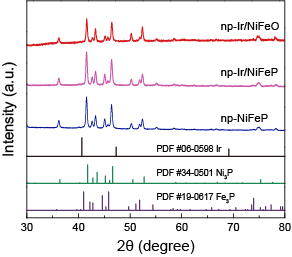


**Supplementary Figure 6.** **XRD characterizations**

XRD profiles, showing the intensity of diffraction versus 2*θ* angle, for np-NiFeP, np-Ir/NiFeP, and np-Ir/NiFeO. The JCPDS profiles of metallic Ir, Ni_3_P, and Fe_3_P are displayed for reference.


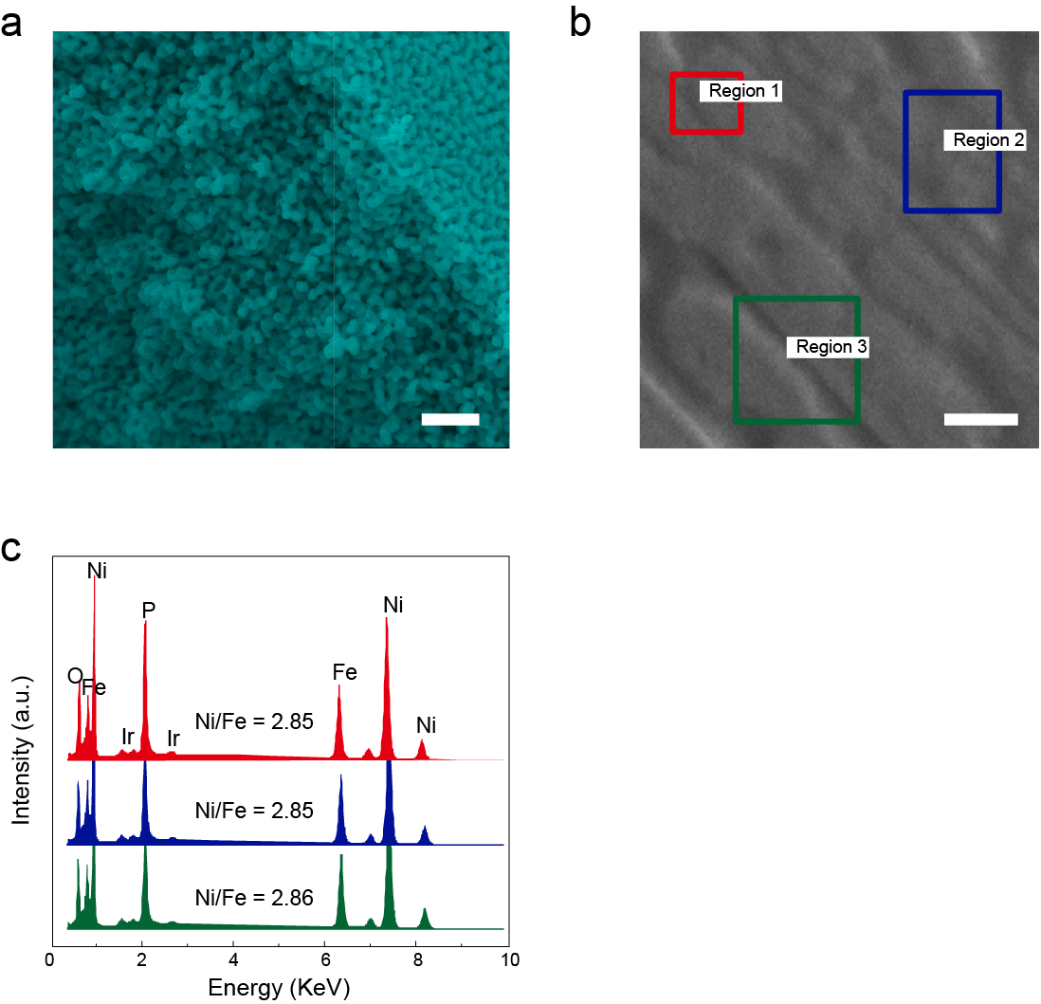


**Supplementary Figure 7.** **SEM characterizations of np-Ir/NiFeP**

(**a**) SEM image of np-Ir/NiFeP. (**b**, **c**) Corresponding EDS spectra. Scale bar: (**a**) 500 nm, (**b**) 10 μm.


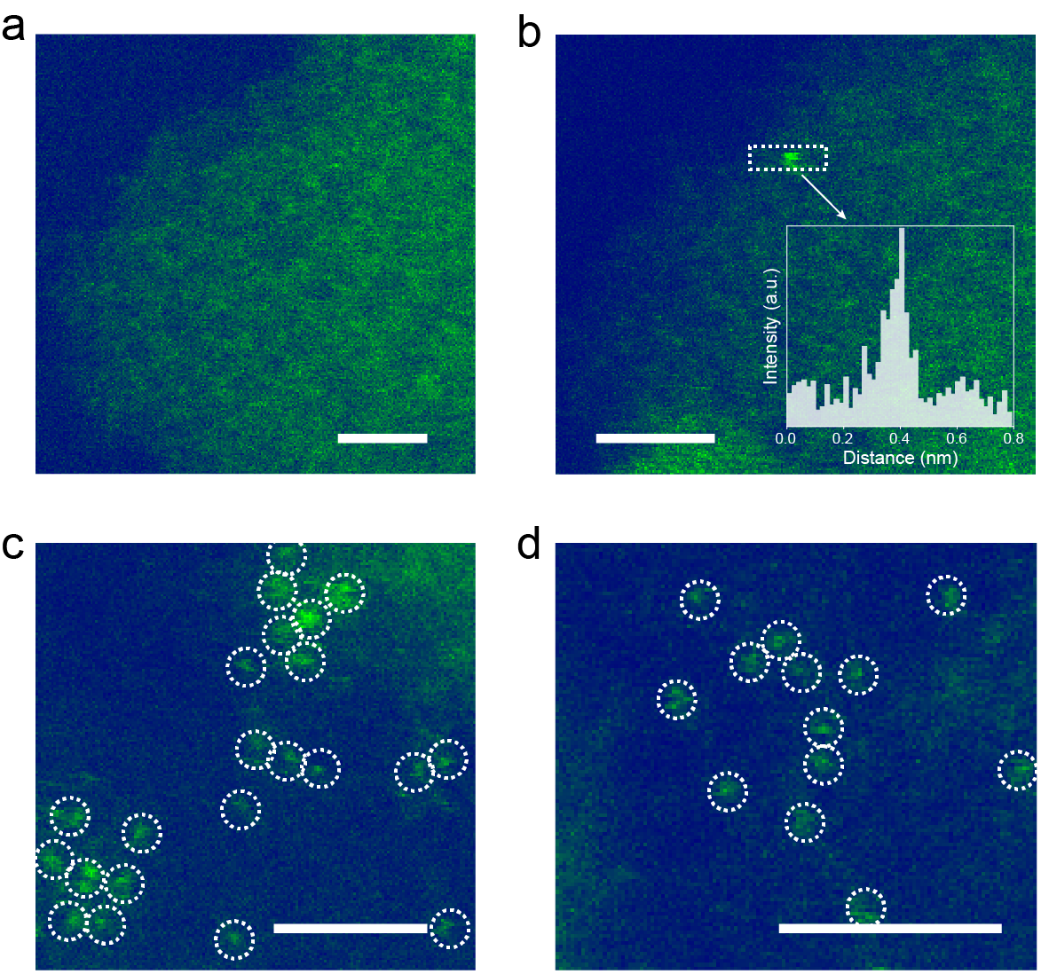


**Supplementary Figure 8.** **HAADF-STEM characterizations of np-Ir/NiFeO**

HAADF-STEM images, showing the surface of Ni(Fe) (oxy)hydroxides without (**a**) and with (**b**) isolated Ir atoms doping. Inset of (b) shows the line-scanning intensity profile. (**c**, **d**) The HAADF-STEM images of np-Ir/NiFeO clearly show that the isolated Ir atoms randomly dispersed on the Ni(Fe) (oxy)hydroxides. The isolated Ir atoms were highlighted by the white circles. Scale bar: (**a**-**d**) 1 nm.


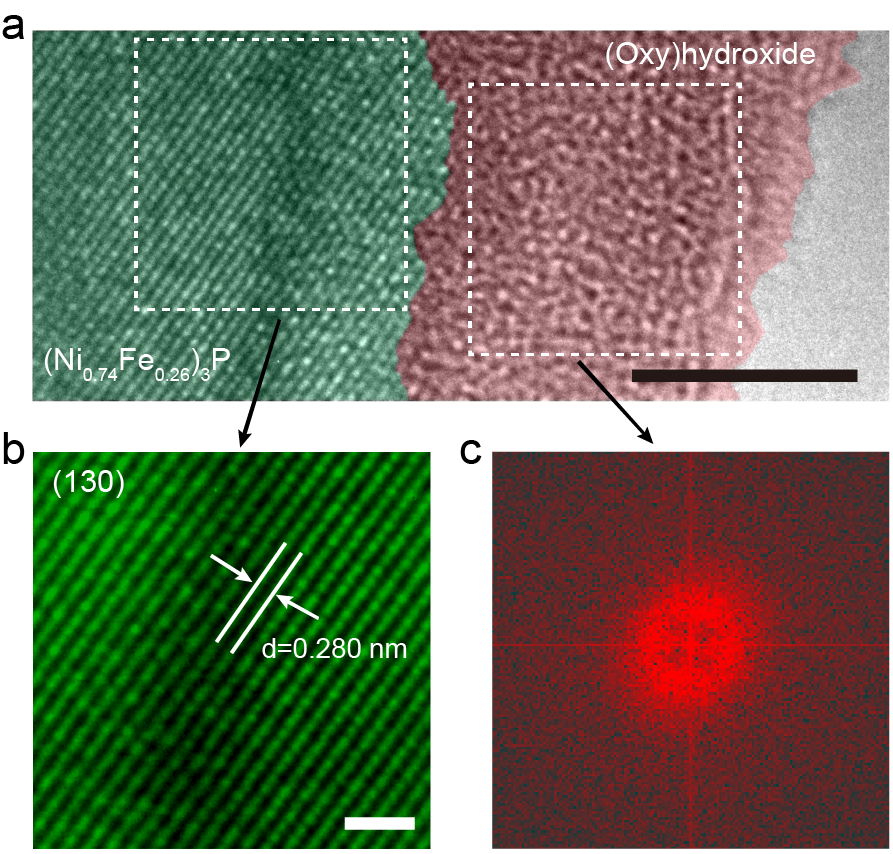


**Supplementary Figure 9.** **HRTEM characterizations of np-Ir/NiFeO**

(**a**) HRTEM image, showing the interface of as-prepared np-Ir/NiFeO. (**b**) The corresponding FFT filtered HRTEM image. (**c**) The corresponding FFT patterns. Scale bar: (**a**) 5 nm, (**b**) 1 nm.


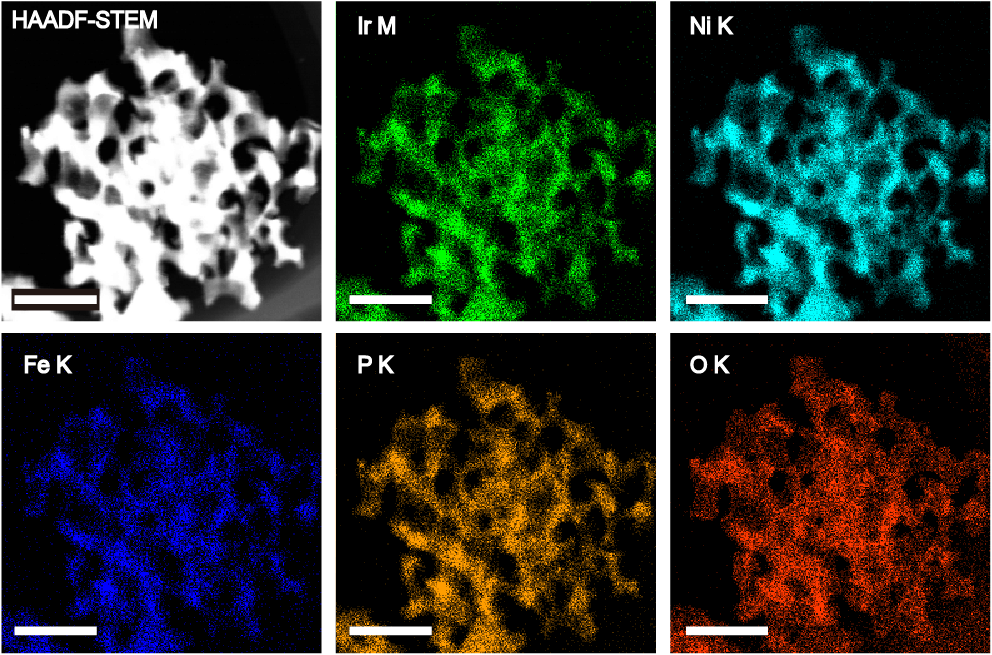


**Supplementary Figure 10. Elemental mapping of np-Ir/NiFeO**

HAADF-STEM image and corresponding EDS elemental mapping, showing the uniformly distribution of Ir, Ni, Fe, P, and O on the whole sample. Scale bar: 200 nm.


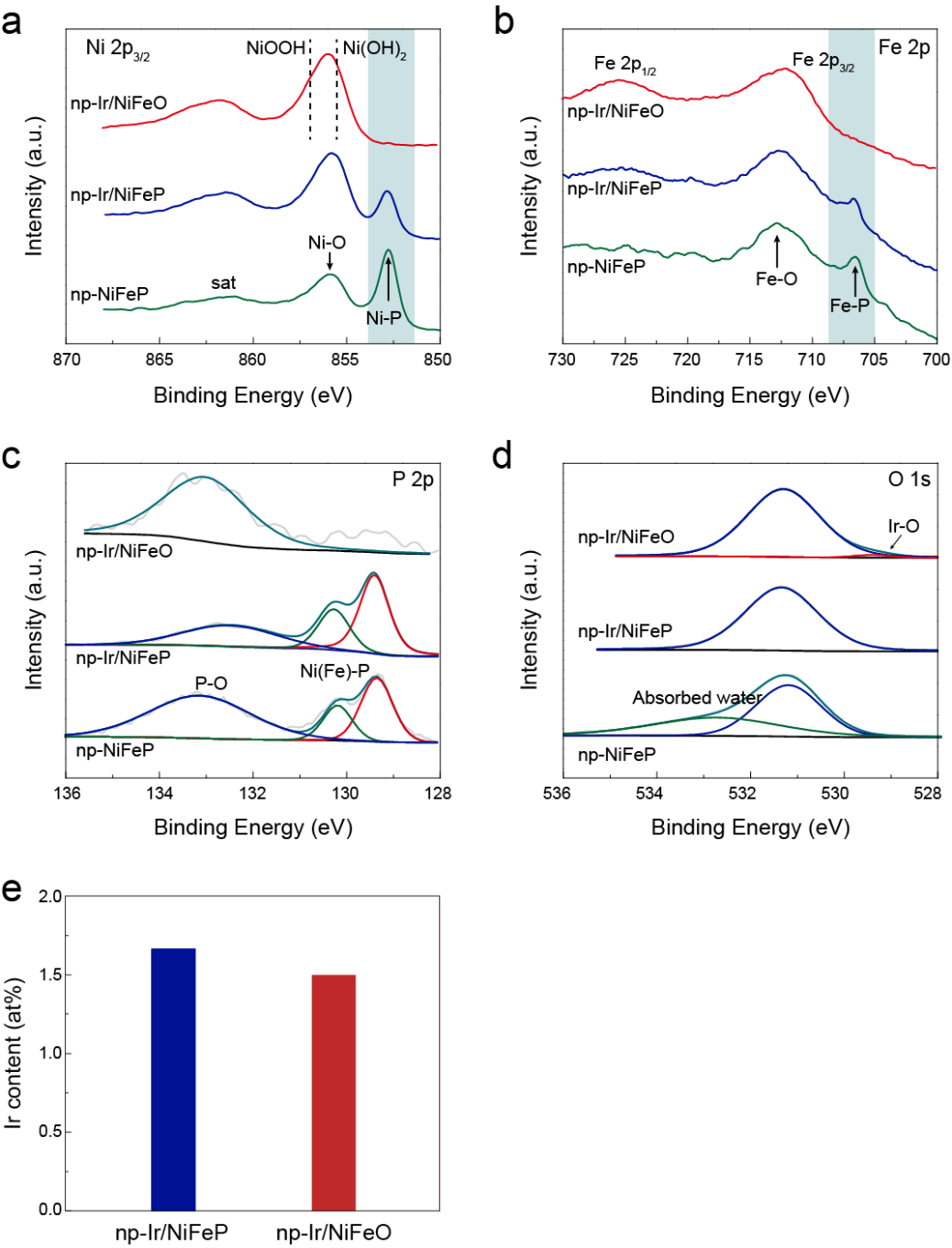


**Supplementary Figure 11.** **XPS** **characterizations**

XPS spectra of np-NiFeP, np-Ir/NiFeP, and np-Ir/NiFeO. (**a**) Ni 2*p*, (**b**) Fe 2*p*, (**c**) P 2*p*, (**d**) O 1*s*. (**e**) Ir content of np-Ir/NiFeP and np-Ir/NiFeO, obtained from XPS results.


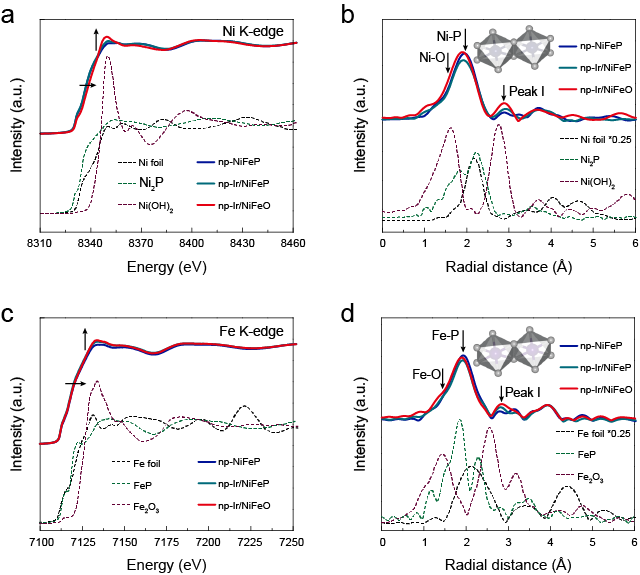


**Supplementary Figure 12.** **XAS** **characterizations**

(**a**, **c**) XANES results of np-NiFeP, np-Ir/NiFeP, and np-Ir/NiFeO at Ni and Fe K-edges. (**b**, **d**) Corresponding FT-EXAFS results of np-NiFeP, np-Ir/NiFeP, and np-Ir/NiFeO. Inset of (b, d) show the schematics of the compositions in Ni(Fe)OOH. Peak I at ~2.81 Å is assigned to the radial distances of Ni-Ni (Fe-Fe).


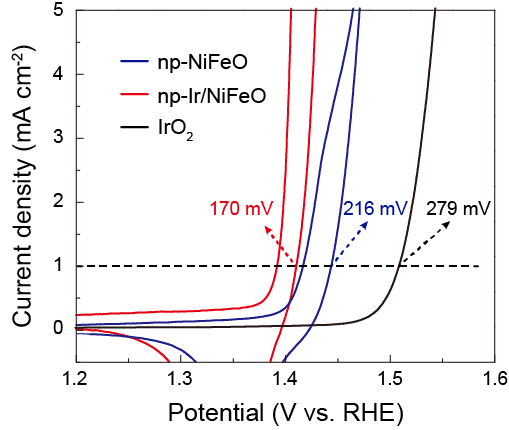


**Supplementary Figure 13. OER polarization curves**

The enlarge OER polarization curves, showing the onset overpotential of np-NiFeO, np-Ir/NiFeO, and IrO_2_. In order to minimize the masking effect of redox couple, we also choose cathodic sweep to compare the onset overpotential.


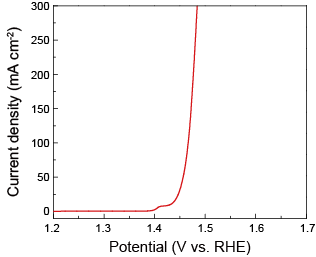


**Supplementary Figure 14. OER performance**

OER polarization curves of np-Ir/NiFeO at high current density.


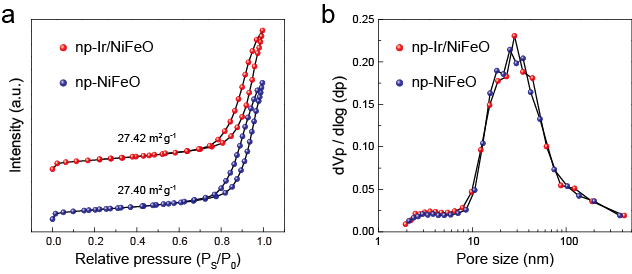


**Supplementary Figure 15. BET characterizations**

(**a**) N_2_ adsorbing-desorbing isotherm curves of np-NiFeO and np-Ir/NiFeO. (**b**) The pore size distribution of np-NiFeO and np-Ir/NiFeO.


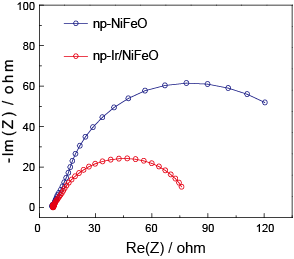


**Supplementary Figure 16. Electrochemical impedance spectroscopy analyses**

Nyquist plots of np-NiFeO and np-Ir/NiFeO at 1.47 V versus RHE.


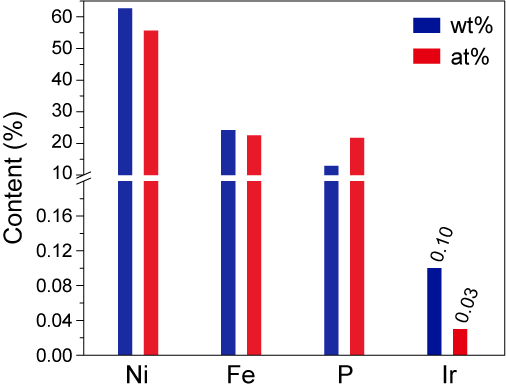


**Supplementary Figure 17. ICP-OES analyses**


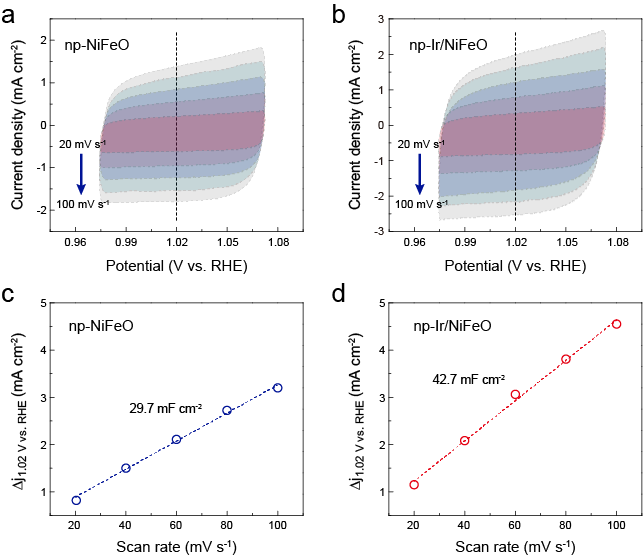


**Supplementary Figure 18.** **Double-layer capacitance analyses**

CVs of (**a**) np-NiFeO and (**b**) np-Ir/NiFeO. These CVs were performed at various scan rates (20, 40, 60, 80, and 100 mV s^-1^). (**c**, **d**) The plots of current densities against scan rates. ∆j is the difference between anodic and cathodic current densities at a potential of 1.02 V versus RHE. The line slopes are equivalent to twice of the electrochemical double-layer capacitance.


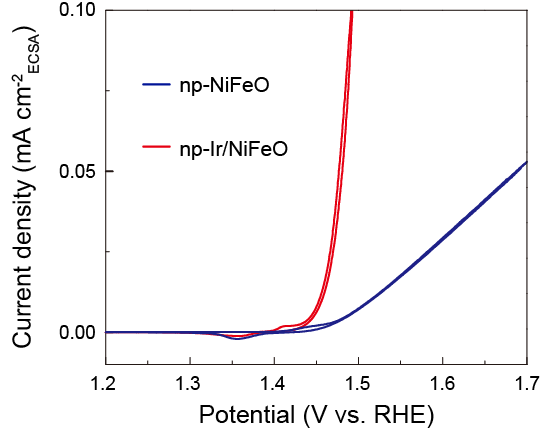


**Supplementary Figure 19.** **Polarization curves**

Polarization curves of different samples normalized by the electrochemical active surface area (ECSA).


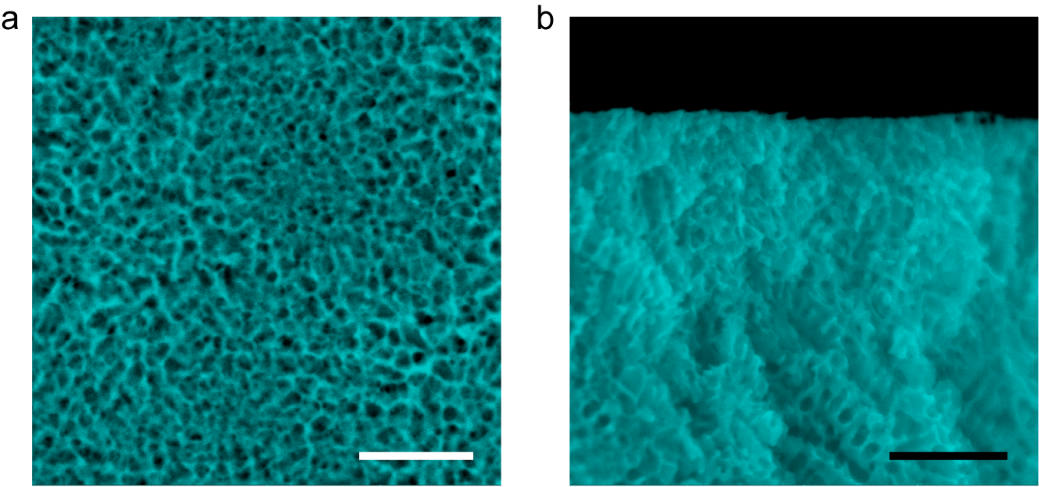


**Supplementary Figure 20.** **SEM** **characterizations after long-time operation**

(**a**) SEM image and (**b**) cross-section SEM image of np-Ir/NiFeO after long-time operation. Scale bar: (**a**, **b**) 500 nm.


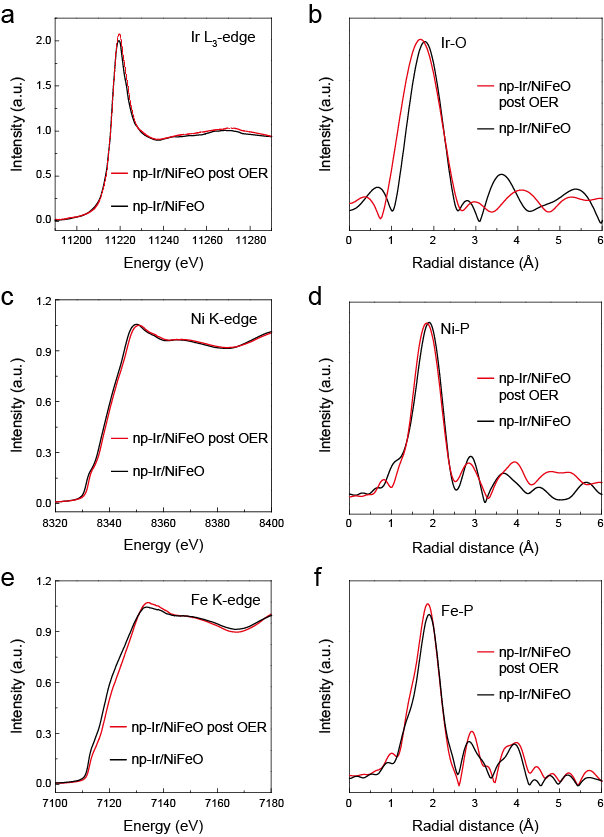


**Supplementary Figure 21. XAS characterizations after long-time operation**

(**a**, **c**, **e**) XANES spectra at Ir L_3_-, Ni K-, and Fe K-edges. (**b**, **d**, **f**) Corresponding FT-EXAFS spectra at Ir L_3_-, Ni K-, and Fe K-edges.


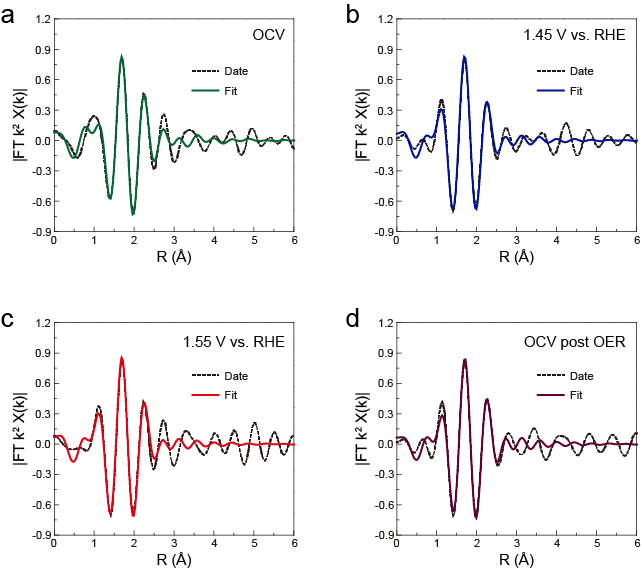


**Supplementary Figure 22. Fitting results in R space**

The R space fitting curves of np-Ir/NiFeO at OCV (**a**), 1.45 V versus RHE (**b**), 1.55 V versus RHE (**c**), and OCV post OER (**d**).


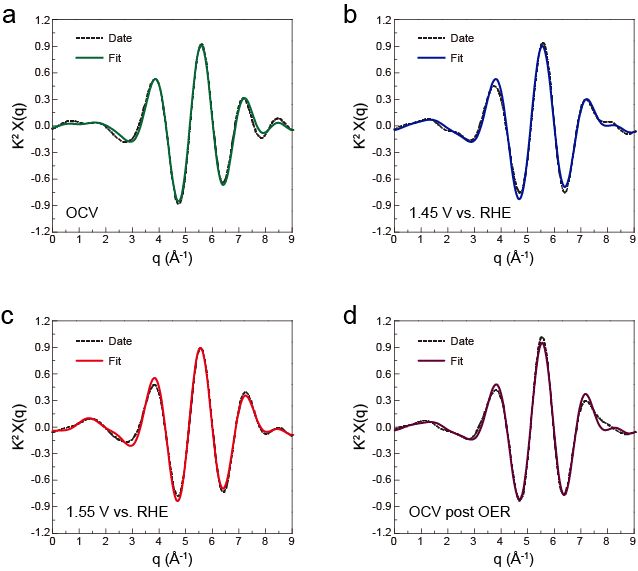


**Supplementary Figure 23. Fitting results in k space**

The k space fitting curves of np-Ir/NiFeO at OCV (**a**), 1.45 V versus RHE (**b**), 1.55 V versus RHE (**c**), and OCV post OER (**d**).


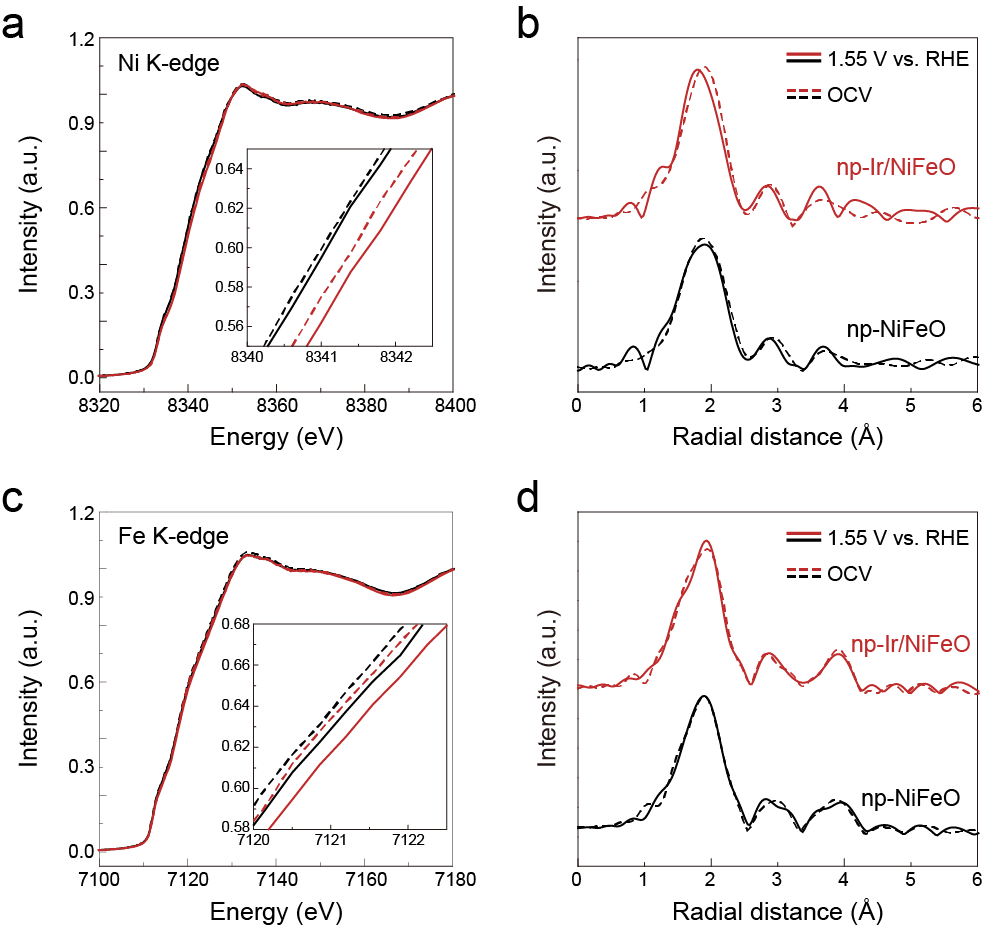


**Supplementary Figure 24. Operando XAS results of np-Ir/NiFeO and np-NiFeO**

(**a**, **b**) Ni K-edge XANES and FT-EXAFS spectra of np-Ir/NiFeO and np-NiFeO at OCV and 1.55 V versus RHE in an O_2_-saturated 1.0 M KOH solution. (**c**, **d**) Fe K-edge XANES and FT-EXAFS spectra of np-Ir/NiFeO and np-NiFeO at OCV and 1.55 V versus RHE in an O_2_-saturated 1 M KOH solution.


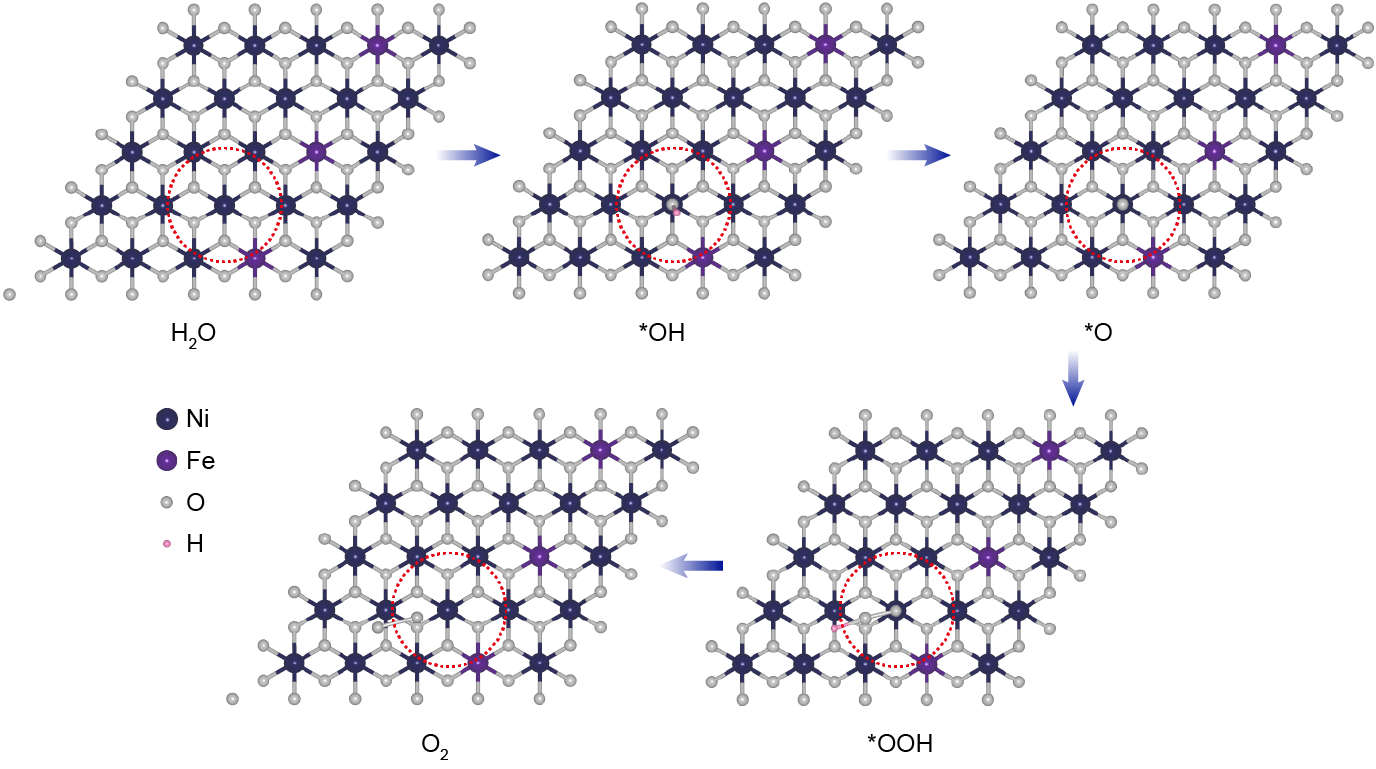


**Supplementary Figure 25. DFT calculations at Ni sites on NiFeO**

The atomic model of NiFeO and the proposed OER pathway on Ni sites.


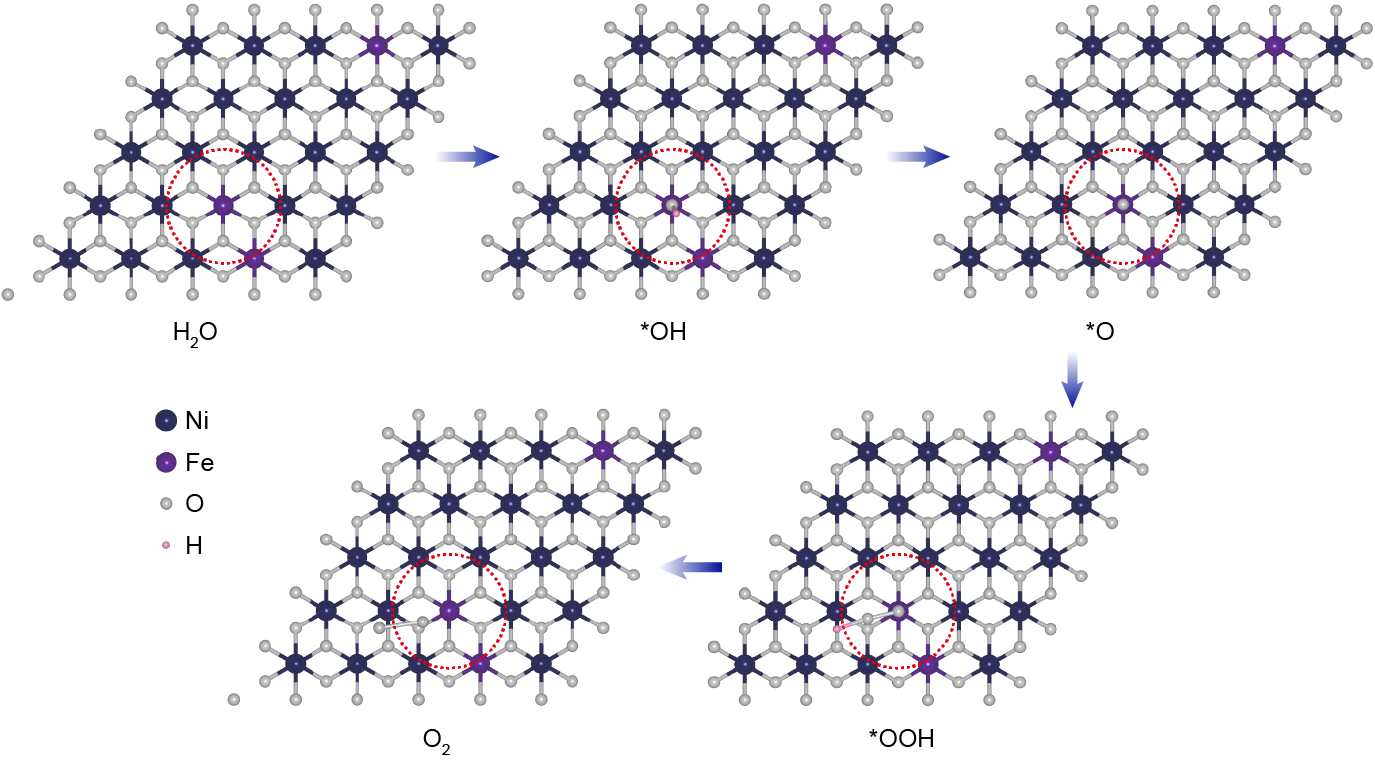


**Supplementary Figure 26. DFT calculations at Fe sites on NiFeO**

The atomic model of NiFeO and the proposed OER pathway on Fe sites.


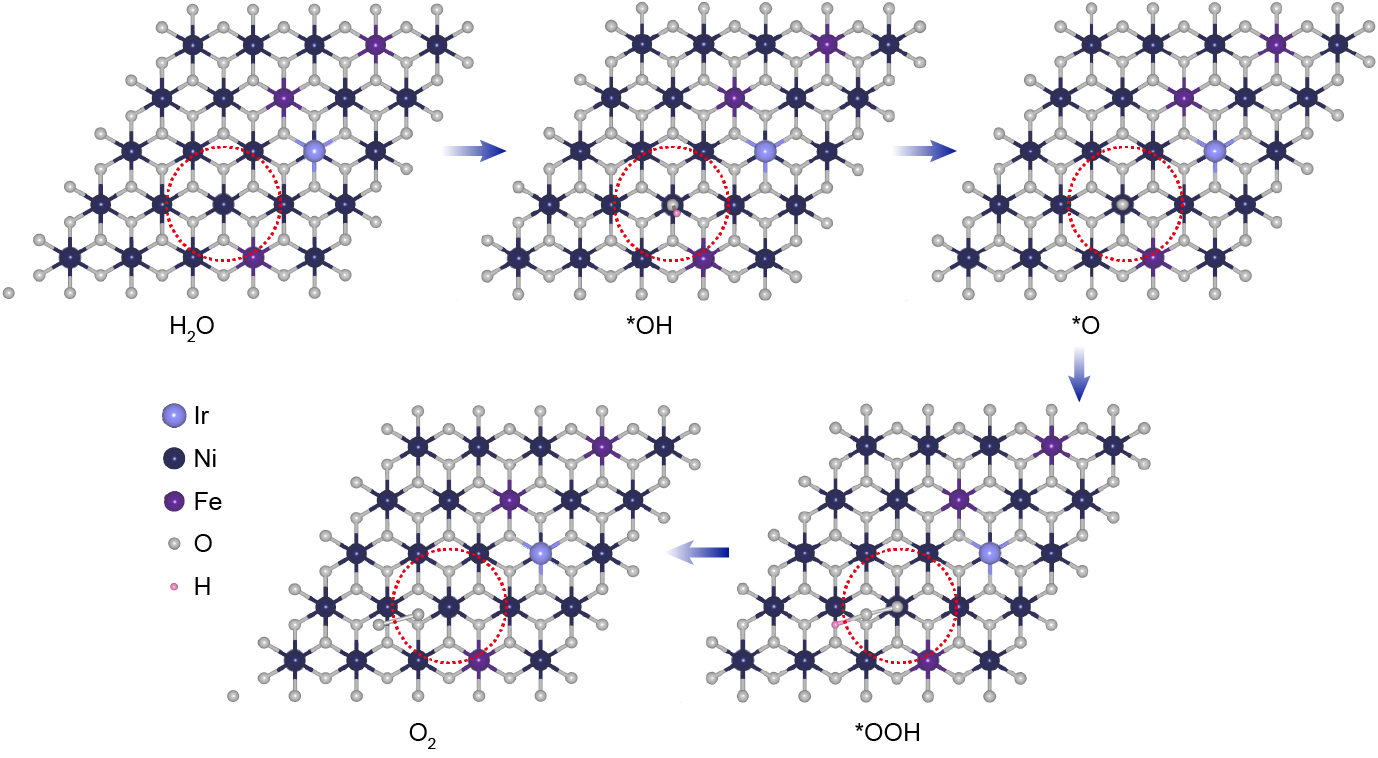


**Supplementary Figure 27. DFT calculations at Ni sites on Ir/NiFeO**

The atomic model of Ir/NiFeO and the proposed OER pathway on Ni sites.


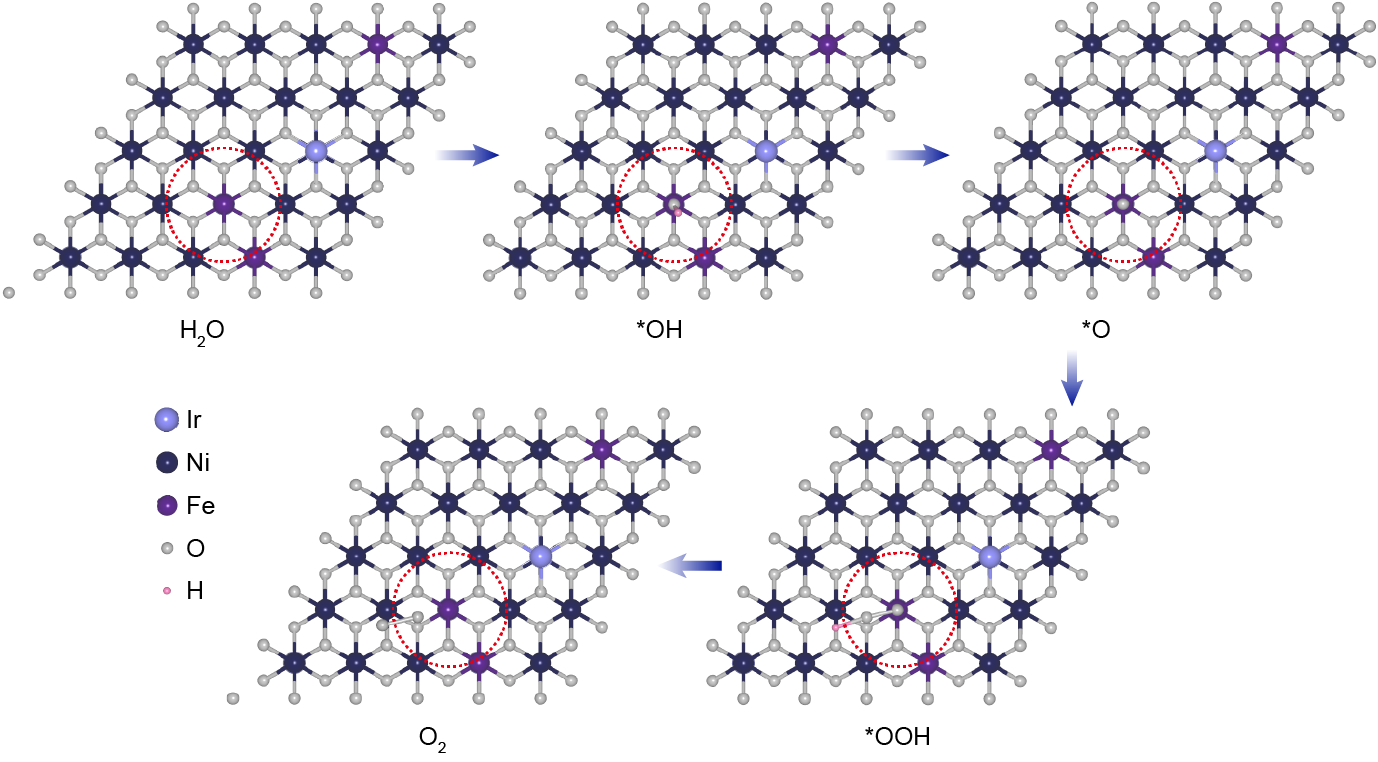


**Supplementary Figure 28. DFT calculations at Fe sites on Ir/NiFeO**

The atomic model of Ir/NiFeO and the proposed OER pathway on Fe sites.


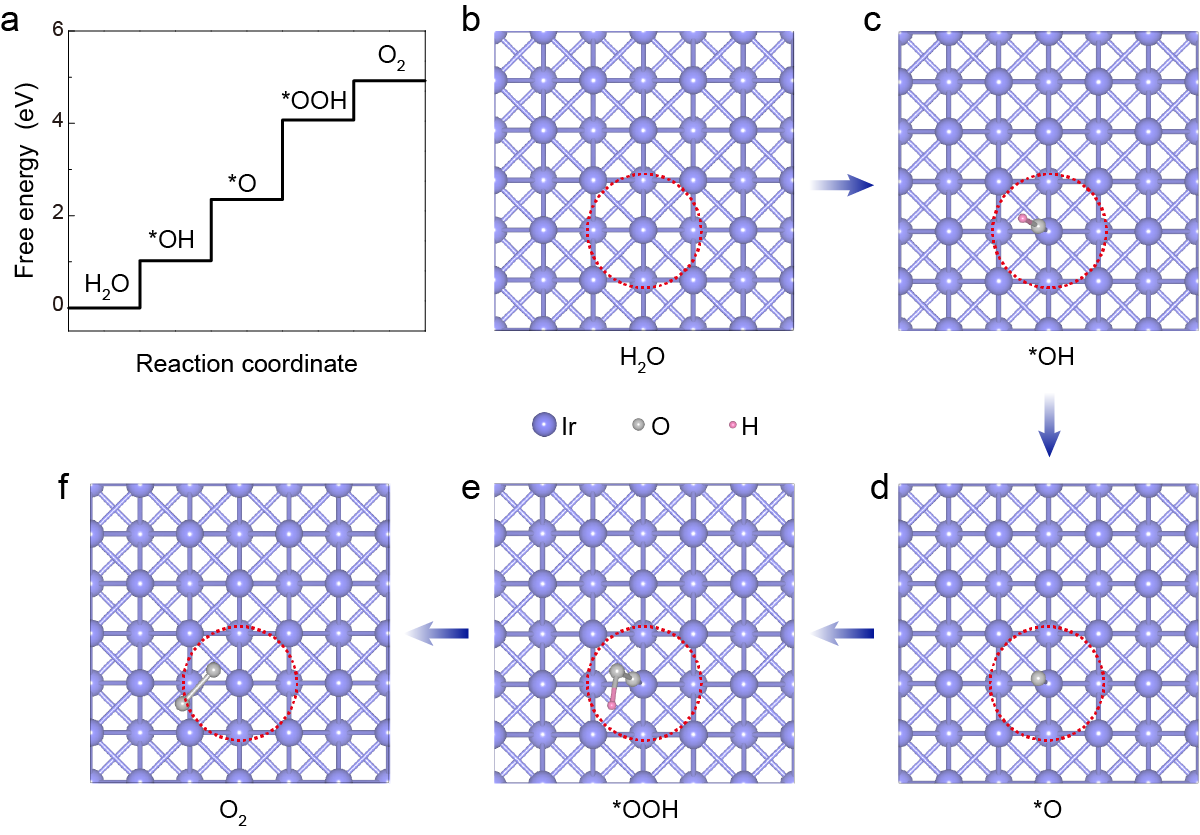


**Supplementary Figure 29. DFT calculations at Ir sites on Ir (001)**

(**a**) Calculated Gibbs free energy profiles of OER at Ir sites on Ir (001). (**b-f**) The atomic model of Ir (001) and the proposed OER pathway on Ir sites.

**Supplementary Tables**

**Supplementary Table 1.** Comparison of mass activity at overpotential of 250 mV of np-Ir/NiFeO with recently reported Ir-based catalysts.

| Catalysts | electrolyte | Mass activity (A mg^-1^) | Ref. |
| --- | --- | --- | --- |
| np-Ir/NiFeO | 1.0 M KOH | 39.32 | This work |
| IrO_2_ | 1.0 M KOH | 0.30 | This work |
| CoIr-0.2 | 1.0 M KOH | About 5.43 | 1 |
| np-Ir_70_Ni_15_Co_15_ | 1.0 M KOH | About 0.23 | 2 |
| 3D Ir | 1.0 M KOH | About 1.91 | 3 |
| IrNi_2_-C | 0.1 M KOH | About 0.13 | 4 |
| IrO_2_/CNT | 1.0 M KOH | About 1.25 | 5 |
| IrNi NCs | 0.1 M KOH | About 1.16 | 6 |

**Supplementary Table 2.** Comparison of overpotential at 10 mA cm^-2^ and Tafel slope of np-Ir/NiFeO with recently reported single-atom catalysts and bulk catalysts.

| Catalysts | | Electrolyte | η@10 mA cm^-2^ (mV) | Tafel slope (mV dec^-1^) | Ref. |
| --- | --- | --- | --- | --- | --- |
| Single-atom catalysts | np-Ir/NiFeO | 1.0 M KOH | 197 | 29.6 | This work |
|  | np-NiFeO | 1.0 M KOH | 260 | 51.6 | This work |
|  | IrO_2_ | 1.0 M KOH | 336 | 66.2 | This work |
|  | CoIr-0.2 | 1.0 M KOH | 235 | 70.2 | 1 |
|  | w-Ni(OH)_2_ | 1.0 M KOH | 237 | 33 | 7 |
|  | S\|NiNx-PC/EG | 1.0 M KOH | 280 | 45 | 8 |
|  | Ru/CoFe-LDHs | 1.0 M KOH | 198 | 39 | 9 |
| Bulk catalysts | np-Ir_70_Co_30_ | 1.0 M KOH | 265 | 70.9 | 2 |
|  | np-Ir_70_Ni_30_ | 1.0 M KOH | 226 | 61.2 | 2 |
|  | np-Ir_70_Ni_15_Co_15_ | 1.0 M KOH | 190 | 57.9 | 2 |
|  | IrO_2_/CNT | 1.0 M KOH | 249 | 32 | 5 |
|  | 3D Ir | 1.0 M KOH | 242 | 32.7 | 3 |
|  | Co@Ir/NC | 1.0 M KOH | 280 | 73.8 | 10 |
|  | np-(Co_0.52_Fe_0.48_)_2_P | 1.0 M KOH | 270 | 30 | 11 |
|  | Co_3_Ni_1_P | 1.0 M KOH | 281 | 66.5 | 12 |
|  | E-CoFe LDH | 1.0 M KOH | 300 | 41 | 13 |
|  | Ni_6/7_Fe_1/7_S_2_ | 1.0 M KOH | 350 | 69 | 14 |
|  | Ni_6/7_Fe_1/7_-OH-6 | 1.0 M KOH | 270 | 40 | 14 |
|  | Ni_2_Fe_1_-O | 1.0 M KOH | 244 | 39 | 15 |
|  | NiVlr-LDH | 1.0 M KOH | 180 | 38 | 16 |
|  | a-LNF(t-d) | 1.0 M KOH | 249 | 36 | 17 |
|  | Fe-Co-P nanoboxes | 1.0 M KOH | 269 | 31 | 18 |

**Supplementary Table 3.** Fitting parameters of FT-EXAFS spectra of np-Ir/NiFeO catalyst under different conditions (CN: coordination number; σ^2^: Debye-Waller factor) ^[19]^.

| *Sample* | *Shell* | *CN* | *R (Å)* | *σ^2^ (Å^2^)* | *R-factor* |
| --- | --- | --- | --- | --- | --- |
| Ex-situ | Ir-O1 | 3.8 | 2.116 | 0.006 | 0.019 |
|  | Ir-O2 | 2.1 | 2.617 | 0.006 |  |
| *Sample* | ***Shell*** | ***CN*** | ***R (Å)*** | ***σ^2^ (Å^2^)*** | ***R-factor*** |
| OCV | Ir-O1 | 3.9 | 2.093 | 0.007 | 0.013 |
|  | Ir-O2 | 2.9 | 2.594 | 0.007 |  |
| *Sample* | ***Shell*** | ***CN*** | ***R (Å)*** | ***σ^2^ (Å^2^)*** | ***R-factor*** |
| 1.45 V versus RHE | Ir-O1 | 4.5 | 2.035 | 0.010 | 0.014 |
|  | Ir-O2 | 4.5 | 2.536 | 0.010 |  |
| *Sample* | ***Shell*** | ***CN*** | ***R (Å)*** | ***σ^2^ (Å^2^)*** | ***R-factor*** |
| 1.55 V versus RHE | Ir-O1 | 4.5 | 2.038 | 0.009 | 0.010 |
|  | Ir-O2 | 4.5 | 2.539 | 0.009 |  |
| *Sample* | ***Shell*** | ***CN*** | ***R (Å)*** | ***σ^2^ (Å^2^)*** | ***R-factor*** |
| OCV post OER | Ir-O1 | 3.8 | 2.050 | 0.007 | 0.013 |
|  | Ir-O2 | 4.0 | 2.552 | 0.007 |  |

**Supplementary Table 4.** Energy barriers for each OER step.

| Sites in catalysts | * → *OH | *OH → *O | *O → *OOH | *OOH →  *OO |
| --- | --- | --- | --- | --- |
| Ni sites in NiFeO | 0.92 eV | 1.62 eV | **1.78 eV** | 0.60 eV |
| Fe sites in NiFeO | 0.91 eV | 1.54 eV | **1.67 eV** | 0.80 eV |
| Ni sites in Ir/NiFeO | 0.75 eV | 1.37 eV | **1.52 eV** | 1.28 eV |
| Fe sites in Ir/NiFeO | 0.71 eV | 1.41 eV | **1.59 eV** | 1.21 eV |
| Ir sites in Ir/NiFeO | 0.61 eV | 1.40 eV | **1.55 eV** | 1.36 eV |
| Ir-O sites in Ir/NiFeO | 0.95 eV | 1.36 eV | **1.42 eV** | 1.19 eV |

**Supplementary Notes**

**Supplementary Note 1: Calculation of the Ir mass activity.**

In order to compare the Ir mass activities of np-Ir/NiFeO and other Ir-based catalysts, their activity values have been normalized to Ir loadings. For np-Ir/NiFeO, the contribution of np-NiFeO has been deducted. The overpotential of 250 mV was selected to evaluate the mass activity. The details are as follows:

$${Supplementary Equation 1: j}_{mass}^{Ir-NiFeO}=\frac{j_{area}^{Ir-NiFeO}-j_{area}^{NiFeO} (mA {cm}^{-2})}{{mass}_{Ir}}=\frac{240.50-7.72 (mA {cm}^{-2})}{0.00592 (mg {cm}^{-2})}=39.32 A {mg}^{-1}$$

Note that as the incorporation of Ir atoms could improve the catalytic activity of support np-NiFeO (including the intrinsic activity and the number of active sites), in the above calculations, the mass-normalized oxygen activity of Ir is the maximum value, and the actual mass-normalized oxygen activity of Ir is lower than this value.

**Supplementary Note 2: Calculation of the electrochemically active surface areas (ECSA).**

The real surface area for OER is calculated from the ECSA, which can be converted from the specific capacitance. The specific capacitance for a flat surface is 40 μF cm^-2^ per cm^2^_ECSA_ ^[20]^.

$${Supplementary Equation 2: A}_{ECSA}^{np-NiFeO}=\frac{14850 mF {cm}^{-2}}{40 \mu F {cm}^{-2} per {cm}_{ECSA}^{2}}=371.25 {cm}_{ECSA}^{2}$$

$${Supplementary Equation 3: A}_{ECSA}^{np-Ir/NiFeO}=\frac{21350 mF {cm}^{-2}}{40 \mu F {cm}^{-2} per {cm}_{ECSA}^{2}}=533.75 {cm}_{ECSA}^{2}$$

**Supplementary Note 3: Density functional theory calculations**

In this work, the scheme developed by Nørskov *et al.* was employed to gain an insight into the thermochemistry mechanism of OER elementary steps ^[21, 22]^. The OER is assumed to involve four elementary reaction steps:

$$Step 1: 2H_{2}O + * \to*OH+ H_{2}O+ H^{+}+e^{-}$$

$$Step 2: *OH + H_{2}O + H^{+}+e^{-} \to*O+ H_{2}O+2(H^{+}+e^{-})$$

$$Step 3: *O+ H_{2}O+2(H^{+}+e^{-}) \to*OOH+ 3(H^{+}+e^{-})$$

$$Step 4: *OOH+ 3\left( H^{+}+e^{-} \right)\to*OO+ 4\left( H^{+}+e^{-} \right)$$

in these equations, the * represents the active site on the catalyst surface.

The Gibbs free energy change is shown in the following equation:

$$Supplementary$$

$$Equation 4: \Delta G=\Delta E+\Delta ZPE-T\Delta S$$

in this equation, ΔE, ΔZPE, and ΔS are the reaction energy, the change in zero point energy, and the change in entropy, respectively. The value of ΔE was determined by the computation of geometrical structures. The values of ΔZPE and ΔS were obtained by employing the computed vibrational frequencies and standard tables for the reactants and products in the gas phase ^[23]^. The entropy of the adsorbed atoms/molecules on the surface active sites of catalysts was assumed to be zero. The temperature dependence of the enthalpy was neglected in the calculations. In this work, ΔG_1_, ΔG_2_, ΔG_3_, and ΔG_4_ are the reaction free energies given in Steps 1-4 of the OER mechanism, respectively. Reaction equation expressions for ΔG_1_, ΔG_2_, ΔG_3_, and ΔG_4_ can be written out as follows:

$$Supplementary$$

$$Equation 5: \Delta G_{1}=E\left( *OH \right)-E\left( * \right)-E_{H2O}+1/2E_{H2}+{(\Delta ZPE-T\Delta S)}_{1}$$

$$Supplementary$$

$$Equation 6: \Delta G_{2}=E\left( *O \right)-E\left( *OH \right)+1/2E_{H2}+{(\Delta ZPE-T\Delta S)}_{2}$$

$$Supplementary$$

$$Equation 7: \Delta G_{3}=E\left( *OOH \right)-E\left( *O \right)-E_{H2O}+1/2E_{H2}+{(\Delta ZPE-T\Delta S)}_{3}$$

$$Supplementary$$

$$Equation 8: \Delta G_{4}=E\left( * \right)-E\left( *OOH \right)+E_{O2}+1/2E_{H2}+{(\Delta ZPE-T\Delta S)}_{4}$$

in these equations, E(*), E(*OH), E(*O), and E(*OOH) are the computed energies for the pure surface and the adsorbed surfaces with *OH, *O, and *OOH, respectively. E_H2O_, E_H2_ and E_O2_ are the computed energies of the H_2_O, H_2_ and O_2_ molecules, respectively. The reaction free energy required to form one molecule of O_2_ in the reaction step can be expressed as:

$$Supplementary$$

$$Equation 9: \Delta G_{2H2O\to O2+2H2}$$

$$=4.92 eV =E_{O2}+2E_{H2}-2E_{H2O}+{(\Delta ZPE-T\Delta S)}_{2H2O\to O2+2H2}$$

**Supplementary References**

1. Zhang, Y. et al. Atomic iridium incorporated in cobalt hydroxide for efficient oxygen evolution catalysis in neutral electrolyte. *Adv. Mater.* **30**, 1707522 (2018).

2. Zhao, Y. et al. 3D nanoporous iridium-based alloy microwires for efficient oxygen evolution in acidic media. *Nano Energy.* **59**, 146-153 (2019).

3. Pi, Y. et al. Ultrathin laminar Ir superstructure as highly efficient oxygen evolution electrocatalyst in broad pH range. *Nano Lett.* **16**, 4424-4430 (2016).

4. Pi, Y. et al. Dynamic structure evolution of composition segregated iridium-nickel rhombic dodecahedra toward efficient oxygen evolution electrocatalysis. *ACS Nano* **12**, 7371-7379 (2018).

5. Guan, J. et al. Synthesis and demonstration of subnanometric iridium oxide as highly efficient and robust water oxidation catalyst. *ACS Catal.* **7***,* 5983-5986 (2017).

6. Pi, Y. et al. General formation of monodisperse IrM (M = Ni, Co, Fe) bimetallic nanoclusters as bifunctional electrocatalysts for acidic overall water splitting. *Adv. Funct. Mater.* **27**, 1700886 (2017).

7. Yan, J., Kong, L., Ji, Y. et al. Single atom tungsten doped ultrathin α-Ni(OH)_2_ for enhanced electrocatalytic water oxidation. *Nat. Commun.* **10**, 2149 (2019).

8. Hou, Y., Qiu, M., Kim, M.G. et al. Atomically dispersed nickel-nitrogen-sulfur species anchored on porous carbon nanosheets for efficient water oxidation. *Nat. Commun.* **10**, 1392 (2019).

9. Li, P., Wang, M., Duan, X. et al. Boosting oxygen evolution of single-atomic ruthenium through electronic coupling with cobalt-iron layered double hydroxides. *Nat. Commun.* **10**, 1711 (2019).

10. Li, D. et al. Total water splitting catalyzed by Co@Ir core-shell nanoparticles encapsulated in nitrogen-doped porous carbon derived from meta-organic frameworks. *ACS. Sustain. Chem. Eng.* **6**, 5105-5114 (2018).

11. Tan, Y. et al. Versatile nanoporous bimetallic phosphides towards electrochemical water splitting. *Energy Environ. Sci.* **9**, 2257-2261 (2016).

12. Fu, S. et al. Highly ordered mesoporous bimetallic phosphides as efficient oxygen evolution electrocatalysts. *ACS Energy Lett.* **1**, 792-796 (2016).

13. Zhou, P. et al. Acid-etched layered double hydroxides with rich defects for enhancing the oxygen evolution reaction. *Chem. Commun.* **53**, 11778-11781 (2017).

14. Wang, T. et al. NiFe (oxy) hydroxides derived from NiFe disulfides as an efficient oxygen evolution catalyst for rechargeable Zn-air batteries: the effect of surface S residues. *Adv. Mater.* **30,** 1800757 (2018).

15. Dong, C. et al. Eutectic-derived mesoporous Ni-Fe-O nanowire network catalyzing oxygen evolution and overall water splitting. *Adv. Mater.* **29**, 1701347 (2017).

16. Wang, D. *et al.* Atomic and electronic modulation of self-supported nickel-vanadium layered double hydroxide to accelerate water splitting kinetics. *Nat. Commun.* **10**, 3899 (2019).

17. Chen, G. *et al.* An amorphous nickel-iron-based electrocatalyst with unusual local structures for ultrafast oxygen evolution reaction. *Adv. Mater.* **31**, 1900883 (2019).

18. Zhang, H. *et al.* Intramolecular electronic coupling in porous iron cobalt (oxy)phosphide nanoboxes enhances the electrocatalytic activity for oxygen evolution. *Energy Environ. Sci.* **12**, 3348-3355 (2019).

19. Zhang, B. *et al.* Homogeneously dispersed multimetal oxygen-evolving catalysts. *Science* **352**, 333-337 (2016).

20. Kibsgaard, J. *et al.* Designing an improved transition metal phosphide catalyst for hydrogen evolution using experimental and theoretical trends. *Energy Environ. Sci.* **8**, 3022-3029 (2015).

21. Rossmeisl, J. et al. Electrolysis of water on oxide surfaces. *J. Electroanal. Chem.* **607**, 83-89 (2007).

22. Man, I. C. et al. Universality in oxygen evolution electrocatalysis on oxide surfaces. *ChemCatChem*, **3**, 1159-1165 (2011).

23. Lide, D. R. CRC handbook of chemistry and physics, 84th ed., CRC, Boca Raton (2004).
