# Supplementary material for: Dynamic active-site generation of atomic iridium stabilized on nanoporous metal phosphides for water oxidation
Source: Nat Commun. 2020 Jun 1;11:2701. doi: 10.1038/s41467-020-16558-1 (PMC7264278; doi:10.1038/s41467-020-16558-1)
Supplement: Supplementary file 2 — Peer Review [file 41467_2020_16558_MOESM2_ESM.pdf]

Reviewers' Comments

Reviewer #1:

The paper describes a nickel iron oxide material functionalized with atomic iridium species. The material shows very impressive catalytic properties towards water oxidation. The authors used XRD, electron microscopic techniques, XPS, XANES and EXAFS to identify the nature of the iridium species and found it to be atomically dispersed.

The proposed mechanism is supported by computational results. There are several things to consider or clarify in this part.

1. The computational details could be much more detailed. The Hubbard-U is used. Which was the value of the U?
2. It is not clear the Gibbs free energy of the species is calculated. Are experimental solvation energies used for the water and hydroxide that are reacting with the surface, or what values are used to get the G? Are thermochemical corrections employed?
3. In Figure 4b the free energies of the final species are very different. It appears odd since it should be the regenerated catalyst and the free energy should be that of the overall reaction.
4. When comparing the mechanism of Ir and Ir-O another thing appears strange. The first step for Ir-O is binding of OH^- and an oxidation leading to IrO(OH). The step is endergonic by 0.3 eV. However the same Ir-O species is a part of the Ir mechanism, where it reacts to form Ir-OOH, a process that is exergonic. Is it not more likely that Ir-O is initiated with the formation of Ir-OOH?
5. Why is the formation of Ir-OOH or IrO(OOH) considered rate limiting? In both cases the free energy of the species is lower than the previous species. Under basic conditions it is likely that the O-O bond can be formed easily and that other steps are limiting the rate.
6. The term "activation energy" is used frequently, but no transition state has been calculated. More correct would be to discuss the free energies of the proposed intermediates and their potential effect on the activation energies.
7. Can a bare Ir on the surface really exist in water? It is likely to be coordinated to water molecules and that should be considered. The initial step would then be Ir(OH2)n -> IrOH(OH2)n-1 + H+.

The activity of the catalyst is very impressive and could be of interest to the readers of NCOMMS, after revision of the mechanistic studies.

Reviewer #2:

In this work, the authors utilize a novel self-reconstruction strategy to prepare a single-atomic iridium catalyst (np-Ir/NiFeO) for efficient oxygen evolution reaction (OER). The np-Ir/NiFeO catalyst exhibited an outstanding OER performance in alkaline solutions, showing an overpotential of 197 mV versus RHE to achieve a current density of 10 mA cm^{-2}, an ultralow Tafel slope of 29.6 mV dec^{-1}, a high mass activity of 39.3 A mg^{-1}, and excellent cycling stability. More importantly, the authors identified the dynamic active site generation of np-Ir/NiFeO under realistic reaction

conditions, namely, the isolated Ir atoms undergo a variation from the single active site to multiple active sites through a deprotonation process, which is responsible for the superior catalytic performance. Meanwhile, the incorporation of Ir atoms could promote the formation of Ni (Fe) oxyhydroxides, further accelerating the reactions. These are significant for the understanding of operando OER enhancement mechanism.

This work not only provides a novel strategy to prepare stable single-atom catalysts, but also put forward a new understanding of the dynamic structure of single-atom catalysts in promoting OER activity. Thus, this work will attract much attention of readers working on energy and materials science. Considering the reasons above, I recommend the acceptance of the manuscript in Nature Communications after minor revisions.

1. The redox couple in Figure 2a is attributed to the conversion between $Ni(OH)_2$ and NiOOH in alkaline electrolytes. The author did not explain how to minimize the masking effect of redox couple in the calculation of the Tafel slope.

2. Onset overpotential is one of the important evaluation criteria applied to characterize the catalytic performances. I think the authors should provide the onset overpotential of different catalysts in this work.

3. In the OER enhancement mechanism part (page 10, line 10-12): The first coordination shell of Ir by considering two backscattering paths, including Ir-O1 (the O ligands and intermediate species) and Ir-O2 (the chemisorption of OH^- and H_2O). The meaning of this sentence is not clear. The authors should further explain their analysis of the Ir coordination shell in this sentence.

4. In DFT simulations, the activation energy of the 4e-process for Ir (001) were calculated in Figure 4b and Supplementary Figure 27 but seems not mentioned in the text. Besides, Fig. 4c is not mentioned in the text.

Reviewer #3:

General comment:

In the manuscript entitled “Dynamic active site generation of single atomic iridium stabilized on nanoporous metal phosphides for accelerating water oxidation, Yongwen Tan and collaborators report on an oxygen evolution catalyst based on single iridium atoms anchored to the amorphous NiFe-based matrix, which shows high activity towards the oxygen evolution reaction (OER) in alkaline media (i.e. KOH 1 M). Authors provide a study by means of electrochemical x-ray absorption spectroscopy, using the XANES and EXAFS approaches, leading them to conclude that-under the anodic operation potentials-the hydroxyl intermediates bound to the iridium sites (claimed in this work as oxygen evolution actives sites) get deprotonated and the thus generated oxo intermediates makes favorable the nucleophilic attack of hydroxide anions. The favorable nucleophilic attack promotes the O-O coupling, which explains the enhanced oxygen evolution activity. This hypothesis was further supported with computational DFT, allowing authors to propose a pathway for the OER on the iridium sites anchored to the NiFeOOH matrix.

In my opinion, the spectro-electrochemical evidence in this manuscript highlights a

very interesting point about the proton-electron transfer reactions (coupled or not) on the iridium sites that lead to O-O coupling, which has been already observed for Ir-based oxygen-evolving molecular catalysts (see for instance JACS 135 (2013) 10837. See also JACS 136 (2014) 10432), but few evidences have been reported for oxide catalysts. The evidence has great value from the fundamental point of view. However, I cannot understand why authors chose alkaline media for the electrochemical and spectro-electrochemical study. In alkaline media, there are many non-noble metal-based oxygen-evolving catalysts with similar or even better performance than iridium-based catalysts (see for instance JACS 135 (2013) 16977. See also ACS Catal. 5 (2015) 5380. See also Science 334 (2011) 1383). Most importantly, I cannot understand why the iridium atoms were anchored to a nickel-iron (oxy)hydroxide matrix.

NiOOH is one of the most active materials for the oxygen evolution reaction in alkaline media, which makes it difficult to understand the results in the manuscript. Unless authors provide conclusive evidence that iridium are the only oxygen-evolving active sites, it is not clear whether the Fe sites are also active (See for instance JACS 137 (2015) 1305. See also ACS Catal. 5 (2015) 5380). The reaction pathway (Fig 4b) was presented to support the hypothesis that iridium is the active site, based on the lower energy barrier to produce oxygen, but this figure is a bit confusing too. It is not clear to me, what Gibbs free energy is plotted in the y-axis of Fig 4b. One would expect 4.92 eV for the theoretical Gibbs free energy needed to transfer the four electrons during the oxygen evolution reaction (see J. Electroanal. Chem. 660 (2011) 254. See also ChemCatChem 3 (2011) 1159), although the energies depicted in Fig 4b are below 1.65 eV, which is thermodynamically not possible for OER. Authors should elaborate further in the discussion of their computational DFT results.

In this manuscript, authors propose that the enhanced nucleophilic attach of hydroxide anions over the oxo intermediate bound to iridium will lead to the O-O coupling and this explains the enhanced oxygen evolution reaction activity of the iridium-doped NiFeOOH. However, nucleophilic attack over the oxo intermediate is unlikely because its rich electron density (the Coulomb repulsion will make this attack very unlikely). The most accepted pathway toward for O-O coupling is reaction between the oxo intermediate and water from the aqueous electrolyte (see for instance J. Electroanal Chem 607 (2007) 83. See also ChemCatChem 3 (2011) 1159).

Since the above-mentioned points directly affect the work's conclusions, I cannot recommend the publication of this manuscript in Nature Communications.

Specific comments:

1. In the manuscript's introduction, authors states that "A state-of-the-art-catalyst with low overpotential and long with low overpotential and long-term stability is still challenging for industrial applications". This is true for acid-based oxygen evolution but not for the alkaline-based (i.e. the relevant case to the manuscript). In fact, there are commercially available (alkaline-based) electrolysers, operating with non-precious metals and having acceptably good performance. I would recommend to authors to rephrase this statement to be relevant to alkaline-based oxygen evolution.

2. In the discussion of the electrochemical performance (p. 8), it is indicated that the

current density up to 300 mA / cm^2 could be due to large specific surface area. It is important for authors to discriminate between geometrical effects (i.e. surface roughness) and true catalytic effects. Authors indicate that the catalysts with single iridium atoms anchored depicted ca. 1.5-fold larger pseudo-capacitance than the undoped material, so surface area normalized activity should be presented to show that the Ir doping has a true catalytic effect.
3. There is a typo in p. 8: hydrogen bubbles cannot be present at 1.48 V vs. RHE.
4. To estimate the mass-normalized oxygen activity, authors are assuming that iridium is the only oxygen evolving sites, which is not totally clear (see the above discussion). To report this, authors should show that NiFe is not electrocatalytically active.

Response to Reviewers' Comments

At first, we sincerely thank all Reviewers for giving us the valuable and thoughtful comments to improve the quality of this manuscript. According to the Reviewers' suggestions, we have performed additional experiments and calculations in order to make following points more clearly. Below we provide point-by-point replies to the Reviewers' comments.

Reviewer #1:
The paper describes a nickel iron oxide material functionalized with atomic iridium species. The material shows very impressive catalytic properties towards water oxidation. The authors used XRD, electron microscopic techniques, XPS, XANES and EXAFS to identify the nature of the iridium species and found it to be atomically dispersed. The proposed mechanism is supported by computational results. There are several things to consider or clarify in this part.

Reply: We thank the Reviewer for the positive remarks and for many constructive suggestions to help us further improve our manuscript. Following the comments, we have carefully revised our manuscript and the details are listed below.

1. The computational details could be much more detailed. The Hubbard-U is used. Which was the value of the U?

Reply: We thank the Reviewer for the helpful suggestions. According to the suggestions, we have added more detailed description about the computation in the revised manuscript (**Methods**) and the revised Supplementary Information (**Supplementary Note 3** and **Supplementary Table 4**).

In this work, the value of U is 3.8 for Ni, 4.3 for Fe, and 0 for Ir (no U correction), which is selected according to the previous literatures (Ref. 55).

Ref. 55: Bi, Y. et al. Understanding the incorporating effect of Co^{2+}/Co^{3+} in NiFe layered double hydroxide for electrocatalytic oxygen evolution reaction. *J. Catal.* **358**, 100-107 (2018).

2. It is not clear the Gibbs free energy of the species is calculated. Are experimental solvation energies used for the water and hydroxide that are reacting with the surface, or what values are used to get the G? Are thermochemical corrections employed?

Reply: We are grateful for these questions. Following these questions, we have added **Supplementary Note 3** in the revised Supplementary Information to further explain our calculations about the Gibbs free energy of the species. For your convenience, we added these discussions as follows:

In this work, the scheme developed by Nørskov *et al.* was employed to gain an insight into the thermochemistry mechanism of OER elementary steps [21, 22]. The OER is assumed to involve four elementary reaction steps:

Step 1: $2H_2O + * \rightarrow *OH + H_2O + H^+ + e^-$

Step 2: $*OH + H_2O + H^+ + e^- \rightarrow *O + H_2O + 2(H^+ + e^-)$

Step 3: $*O + H_2O + 2(H^+ + e^-) \rightarrow *OOH + 3(H^+ + e^-)$

Step 4: $*OOH + 3(H^+ + e^-) \rightarrow *OO + 4(H^+ + e^-)$

in these equations, the * represents the active site on the catalyst surface.

The Gibbs free energy change is shown in the following equation:

Equation 1: $\Delta G = \Delta E + \Delta ZPE - T\Delta S$

in this equation, ΔE, ΔZPE, and ΔS are the reaction energy, the change in zero point energy, and the change in entropy, respectively. The value of ΔE was determined by the computation of geometrical structures. The values of ΔZPE and ΔS were obtained by employing the computed vibrational frequencies and standard tables for the reactants and products in the gas phase. The entropy for the adsorbed atoms/molecules at the surface active site was assumed to be zero. The temperature dependence of the enthalpy was neglected in the calculations. In this work, ΔG_1, ΔG_2, ΔG_3, and ΔG_4 are the reaction free energies given in Steps 1-4 of the OER mechanism, respectively. Reaction equation expressions for ΔG_1, ΔG_2, ΔG_3, and ΔG_4 can be written out as follows:

Equation 2: $\Delta G_1 = E(*OH) - E(*) - E_{H2O} + 1/2E_{H2} + (\Delta ZPE - T\Delta S)_1$

Equation 3: $\Delta G_2 = E(*O) - E(*OH) + 1/2E_{H2} + (\Delta ZPE - T\Delta S)_2$

Equation 4: $\Delta G_3 = E(*OOH) - E(*O) - E_{H2O} + 1/2E_{H2} + (\Delta ZPE - T\Delta S)_3$

Equation 5: $\Delta G_4 = E(*) - E(*OOH) + E_{O2} + 1/2E_{H2} + (\Delta ZPE - T\Delta S)_4$

in these equations, E(*), E(*OH), E(*O), and E(*OOH) are the computed energies for the pure surface and the adsorbed surfaces with *OH, *O, and *OOH, respectively. E_{H2O}, E_{H2} and E_{O2} are the computed energies of the H_2O, H_2 and O_2 molecules, respectively. The reaction free energy required to form one molecule of O_2 in the reaction step can be expressed as:

Equation 6: $\Delta G_{2H2O \rightarrow O2+2H2}$
$= 4.92\ \text{eV} = E_{O2} + 2E_{H2} - 2E_{H2O} + (\Delta ZPE - T\Delta S)_{2H2O \rightarrow O2+2H2}$

Ref. 21: Rossmeisl, J. et al. Electrolysis of water on oxide surfaces. *J. Electroanal. Chem.* **607**, 83-89 (2007).

Ref. 22: Man, I. C. et al. Universality in Oxygen Evolution Electrocatalysis on Oxide Surfaces. *ChemCatChem*, **3**, 1159-1165 (2011).

Besides, we do not consider the experimental solvation energies for the water and hydroxide in the calculations part. It is true that considering the experimental solvation energies will make the theoretical results closer to the actual results. In this work, DFT calculation was used to further support our mechanism derived from *operando* X-ray absorption spectroscopy results. On the one hand, it confirmed that the d-electron domination of surface Ni and Fe atoms were optimize after the introduction of Ir atoms. On the other hand, it proved the Ni and Fe sites is activated by the introduction of Ir atoms, thus transforming to the more favorable OER active sites. Therefore, we did not pursue very precise theoretical results but pay more attention to the change of active sites on the surface of catalysts before and after Ir doping.

3. In Figure 4b the free energies of the final species are very different. It appears odd since it should be the regenerated catalyst and the free energy should be that of the overall reaction.

Reply: We thank the Reviewer for this comment. We have revised relevant calculations and figures in the revised manuscript (**Fig. 4b**). For your convenience, this change is also shown in **Fig. R1**. For all OER active sites, the free energy of the final species is 4.92 eV, which is also the free energy of the overall reaction (please see ***comment 2*** for detail).

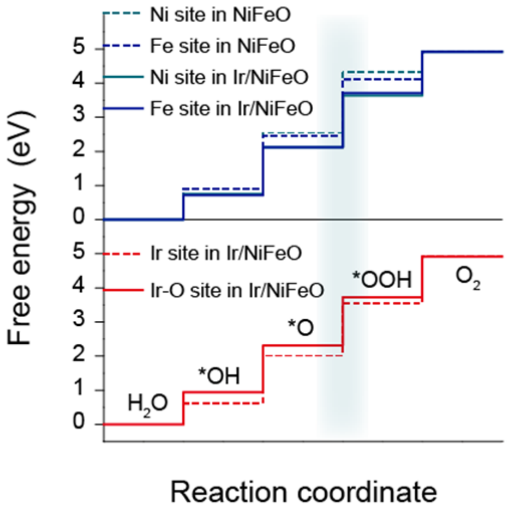

Figure R1 DFT calculations

Calculated free energy diagram of the OER. The blue box step is the rate determining step.

4. When comparing the mechanism of Ir and Ir-O another thing appears strange. The first step for Ir-O is binding of OH^- and an oxidation leading to IrO(OH). The step is endergonic by 0.3 eV. However the same Ir-O species is a part of the Ir mechanism, where it reacts to form Ir-OOH, a process that is exergonic. Is it not more likely that Ir-O is initiated with the formation of Ir-OOH?

Reply: We appreciate the Reviewer for these nice questions. Under high applied potential, OH^- adsorption and deprotonation on the Ir sites was observed by the *operando* X-ray absorption spectroscopy measurement. It is possible that Ir-O species could directly react to form Ir-OOH. Simultaneously, other adsorbed OH^- could deprotonate to form new adsorbed O. In the oxygen evolution cycle, the stable existence of Ir-O species was proved by our experiments (**Fig. 3a-c** in the revised manuscript), as evidenced by the increase of CN_{O1} under realistic OER conditions (**Fig. R2**). Thus, the effect of Ir-O species on the coupling of other O in the case of multiple oxygen adsorptions should be considered and computed.

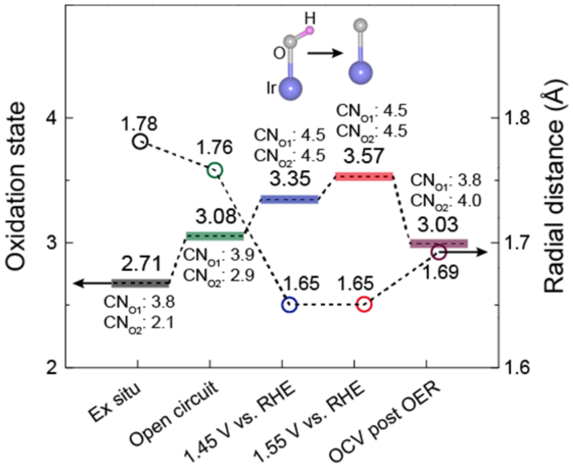

Figure R2 XAS analysis

The fitted oxidation states from the white line intensity analysis, the variation of Ir-O bond, and the FT-EXAFS curve-fitting analysis.

5. Why is the formation of Ir-OOH or IrO(OOH) considered rate limiting? In both cases the free energy of the species is lower than the previous species. Under basic conditions it is likely that the O-O bond can be formed easily and that other steps are limiting the rate.

Reply: We appreciate the Reviewer for these comments. After carefully consider our model and relevant literature, we have revised our calculations (**Fig. 4b, c** in the revised manuscript). For both np-NiFeO and np-Ir/NiFeO, the OER rate determining step was found to be the formation of *OOH group from *O group, as also shown in **Table R1** (**Supplementary Table 4** in the revised Supplementary Information).

Table R1. Energy barriers for each OER step.

Sites in catalysts	* → *OH	*OH → *O	*O → *OOH	*OOH → *OO
**Ni sites in NiFeO**	0.92 eV	1.62 eV	**1.78 eV**	0.60 eV
**Fe sites in NiFeO**	0.91 eV	1.54 eV	**1.67 eV**	0.80 eV
**Ni sites in Ir/NiFeO**	0.75 eV	1.37 eV	**1.52 eV**	1.28 eV
**Fe sites in Ir/NiFeO**	0.71 eV	1.41 eV	**1.59 eV**	1.21 eV
**Ir sites in Ir/NiFeO**	0.61 eV	1.40 eV	**1.55 eV**	1.36 eV
**Ir-O sites in Ir/NiFeO**	0.95 eV	1.36 eV	**1.42 eV**	1.19 eV

6. The term "activation energy" is used frequently, but no transition state has been calculated. More correct would be to discuss the free energies of the proposed intermediates and their potential effect on the activation energies.

Reply: We are grateful to the reviewer for the very helpful suggestion. According to your suggestion, we have revised relevant discussions in the revised manuscript (**Page 14, line 3-17**).

7. Can a bare Ir on the surface really exist in water? It is likely to be coordinated to water molecules and that should be considered. The initial step would then be Ir(OH2)n -> IrOH(OH2)n-1 + H+.

Reply: We are grateful to the reviewer for the valuable comments. We agree with the Reviewer's point that the initial step would then be Ir(OH2)n -> IrOH(OH2)n-1 + H+. Following the comments, we have carefully revised our calculations and the details are listed below (**Fig. 4b-d** in the revised manuscript. **Supplementary Note 3** in the revised Supplementary Information):

In this work, the scheme developed by Nørskov *et al.* was employed to gain an insight into the thermochemistry mechanism of OER elementary steps [21, 22]. The OER is assumed to involve four elementary reaction steps:

Step 1: $2H_2O + * \rightarrow *OH + H_2O + H^+ + e^-$

Step 2: $*OH + H_2O + H^+ + e^- \rightarrow *O + H_2O + 2(H^+ + e^-)$

Step 3: $*O + H_2O + 2(H^+ + e^-) \rightarrow *OOH + 3(H^+ + e^-)$

Step 4: $*OOH + 3(H^+ + e^-) \rightarrow *OO + 4(H^+ + e^-)$

where * represents the active site in the specific model.

Ref. 21: Rossmeisl, J. et al. Electrolysis of water on oxide surfaces. *J. Electroanal. Chem.* **607**, 83-89 (2007).

Ref. 22: Man, I. C. et al. Universality in Oxygen Evolution Electrocatalysis on Oxide Surfaces. *ChemCatChem*, **3**, 1159-1165 (2011).

The activity of the catalyst is very impressive and could be of interest to the readers of NCOMMS, after revision of the mechanistic studies.

Reply: We sincerely appreciate your positive review.

Reviewer #2:
In this work, the authors utilize a novel self-reconstruction strategy to prepare a single-atomic iridium catalyst (np-Ir/NiFeO) for efficient oxygen evolution reaction (OER). The np-Ir/NiFeO catalyst exhibited an outstanding OER performance in alkaline solutions, showing an overpotential of 197 mV versus RHE to achieve a current density of 10 mA cm^{-2}, an ultralow Tafel slope of 29.6 mV dec^{-1}, a high mass activity of 39.3 A mg^{-1}, and excellent cycling stability. More importantly, the authors identified the dynamic active site generation of np-Ir/NiFeO under realistic reaction conditions, namely, the isolated Ir atoms undergo a variation from the single active site to multiple active sites through a deprotonation process, which is responsible for the superior catalytic performance. Meanwhile, the incorporation of Ir atoms could promote the formation of Ni (Fe) oxyhydroxides, further accelerating the reactions. These are significant for the understanding of operando OER enhancement mechanism.
This work not only provides a novel strategy to prepare stable single-atom catalysts, but also put forward a new understanding of the dynamic structure of single-atom catalysts in promoting OER activity. Thus, this work will attract much attention of readers working on energy and materials science. Considering the reasons above, I recommend the acceptance of the manuscript in Nature Communications after minor revisions.

Reply: We thank the Reviewer for the valuable comments and for pointing out the novelty and importance of our work. Following the comments, we have carefully revised our manuscript and the details are listed below.

1. The redox couple in Figure 2a is attributed to the conversion between $Ni(OH)_2$ and NiOOH in alkaline electrolytes. The author did not explain how to minimize the masking effect of redox couple in the calculation of the Tafel slope.

Reply: Thank you for this comment. To minimize the masking effect of redox couple, we derived the Tafel plots and compared the OER activities using the cathodic sweep. We have added this discussion in the revised manuscript (**Methods** in revised manuscript).

2. Onset overpotential is one of the important evaluation criteria applied to

characterize the catalytic performances. I think the authors should provide the onset overpotential of different catalysts in this work.

Reply: We thank the Reviewer for the insightful comments. Following this comment, we have added **Fig. R3** (**Supplementary Fig. 13** in the revised Supplementary Information) and relevant discussions (**Page 8, line 9-12**) about the onset overpotential in the revised manuscript:

The np-Ir/NiFeO shows the highest performance among all samples, requiring overpotential of only 170 and 197 mV to reach a current density of 1(defined as onset overpotential) and 10 mA cm^{-2}, significantly better than those of np-NiFeO and commercial IrO_2.

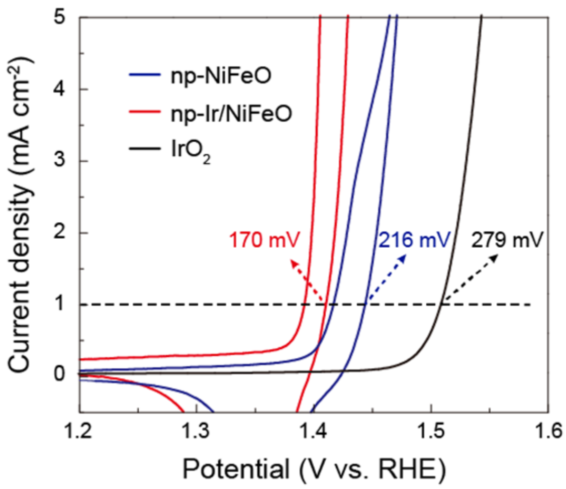

Figure R3 OER polarization curves

The enlarge OER polarization curves, showing the onset overpotential of np-NiFeO, np-Ir/NiFeO, and IrO_2. In order to minimize the masking effect of redox couple, we also choose cathodic sweep to compare the onset overpotential.

3. In the OER enhancement mechanism part (page 10, line 10-12): The first coordination shell of Ir by considering two backscattering paths, including Ir-O1 (the O ligands and intermediate species) and Ir-O2 (the chemisorption of OH^- and H_2O). The meaning of this sentence is not clear. The authors should further explain their analysis of the Ir coordination shell in this sentence.

Reply: Thanks for your suggestion. We have corrected this discussion in the revised manuscript (**Page 10, line 16-19**):

The analysis of Ir coordination shell was carried out by taking two backscattering paths, including Ir-O1 (the O ligands and intermediate species) and Ir-O2 (the chemisorption of OH^- and H_2O).

4. In DFT simulations, the activation energy of the 4e-process for Ir (001) were

calculated in Figure 4b and Supplementary Figure 27 but seems not mentioned in the text. Besides, Fig. 4c is not mentioned in the text.

Reply: We thank the Reviewer for the constructive comment. Following this comment, we have added relevant discussions in the revised manuscript (**Page 14, line 8-11**) and the revised Supplementary Information (**Supplementary Fig. 29**).

Reviewer #3:

General comment:

In the manuscript entitled “Dynamic active site generation of single atomic iridium stabilized on nanoporous metal phosphides for accelerating water oxidation, Yongwen Tan and collaborators report on an oxygen evolution catalyst based on single iridium atoms anchored to the amorphous NiFe-based matrix, which shows high activity towards the oxygen evolution reaction (OER) in alkaline media (i.e. KOH 1 M). Authors provide a study by means of electrochemical x-ray absorption spectroscopy, using the XANES and EXAFS approaches, leading them to conclude that-under the anodic operation potentials-the hydroxyl intermediates bound to the iridium sites (claimed in this work as oxygen evolution actives sites) get deprotonated and the thus generated oxo intermediates makes favorable the nucleophilic attack of hydroxide anions. The favorable nucleophilic attack promotes the O-O coupling, which explains the enhanced oxygen evolution activity. This hypothesis was further supported with computational DFT, allowing authors to propose a pathway for the OER on the iridium sites anchored to the NiFeOOH matrix.

In my opinion, the spectro-electrochemical evidence in this manuscript highlights a very interesting point about the proton-electron transfer reactions (coupled or not) on the iridium sites that lead to O-O coupling, which has been already observed for Ir-based oxygen-evolving molecular catalysts (see for instance JACS 135 (2013) 10837. See also JACS 136 (2014) 10432), but few evidences have been reported for oxide catalysts. The evidence has great value from the fundamental point of view.

Reply: We appreciate for your encouraging and constructive comments. We also thank you for bringing out our attention to the previous important work. In order to further support our experimental results, we have cited these relevant literatures in the revised manuscript:

Ref 46: Hintermair, U. et al. Precursor transformation during molecular oxidation catalysis with organometallic iridium complexes. *J. Am. Chem. Soc.* **135**, 10837-10851 (2013).

Ref 47: Diaz-Morales, O. et al. Electrochemical and spectroelectrochemical characterization of an iridium-based molecular catalyst for water splitting: turnover frequencies, stability, and electrolyte effects. *J. Am. Chem. Soc.* **136**, 10432-10439 (2014).

However, I cannot understand why authors chose alkaline media for the electrochemical and spectro-electrochemical study. In alkaline media, there are many non-noble metal-based oxygen-evolving catalysts with similar or even better performance than iridium-based catalysts (see for instance JACS 135 (2013) 16977. See also ACS Catal. 5 (2015) 5380. See also Science 334 (2011) 1383).

Reply: We are grateful for these comments. We would like to emphasize that one of the primary motivations of this work is to report the self-reconstruction strategy which provide new view in the preparation of stable single-atom catalysts for oxygen evolution reaction. Hence, the selection of support for the anchoring of single atom is very essential for the self-reconstruction strategy. Unfortunately, Ni(Fe)OOH is unstable under acid and neutral conditions, as mentioned in the above literature that the non-noble metal catalysts (including $NiFeO_x$) are not stable in acidic solutions under oxidizing conditions (JACS 135 (2013) 16977). So we chose alkaline media for the electrochemical and spectro-electrochemical study.

Meanwhile, though great efforts have been made to develop the non-noble metal-based oxygen-evolving catalysts. Considering the aim of high energy efficiency, their catalytic performance remains far from satisfactory. Therefore, it is significant to further improve the catalytic performance of these catalysts. In this work, the coupling of trace amounts of Ir and non-noble support enables an inexpensive and highly efficient oxygen production.

In order to further deliver the significance of our work, we have cited the above literature in the revised manuscript:

Ref 11: McCrory, C. C. et al. Benchmarking heterogeneous electrocatalysts for the oxygen evolution reaction. *J. Am. Chem. Soc.* **135**, 16977-16987 (2013).

Ref 12: Diaz-Morales, O. et al. Guidelines for the rational design of Ni-based double hydroxide electrocatalysts for the oxygen evolution reaction. *ACS Catal.* **5**, 5380-5387 (2015).

Ref 13: Suntivich, J. et al. A perovskite oxide optimized for oxygen evolution catalysis from molecular orbital principles. *Science.* **334**, 1383-1385 (2011).

Most importantly, I cannot understand why the iridium atoms were anchored to a nickel-iron (oxy)hydroxide matrix.

Reply: We thank the Reviewer for this question. In the self-reconstruction strategy, NiFeP was selected as support for the anchoring of single-atom Ir. XPS results confirmed the existence of abundant Ni(Fe) oxide and hydroxide on the surface of NiFeP (**Supplementary Fig. 11** in the revised Supplementary Information. *ACS Catal.* **8**, 4408-4419 (2018). DOI: 10.1021/acscatal.7b04466). During the electrochemical single-atom deposition in basic solutions, surface NiFeP will further transform to Ni(Fe) oxide and hydroxide by the partial dissolution of P (*ACS Energy Lett.* **4**, 987-994 (2019). DOI: 10.1021/acsenergylett.9b00382. / *Adv. Mater.* **28**, 2951-2955

(2016). DOI: 10.1002/adma.201505875), manifesting as the break of Ni-P bonds (**Page 7, line 15-18** in the revised manuscript and **Supplementary Fig. 12b** in the revised Supplementary Information). Simultaneously, Ir cations were driven towards the cathode by the applied electric field and deposited on the support by coordinate with O atoms of Ni(Fe) oxide and hydroxide (*Nat. Catal.* **1**, 985-992 (2018). DOI: 10.1038/s41929-018-0195-1). After self-reconstruction, Ni(Fe) oxide and hydroxide could transfer to Ni(Fe) oxyhydroxide, thus further stabilize single-atom Ir. This was evidenced by the increase of the intensity of Ir-O shell in the FT-EXAFS spectrum, indicating the increase of coordinated O atoms (**Fig. 1h** in the revised manuscript).

Moreover, we performed FT-EXAFS curve-fitting analysis to further identify the atomic coordination structure of Ir atoms (as shown in **Fig. R4**). It is clear that Ir atoms are indeed coordinated with O ligands on Ni(Fe) oxyhydroxide, in accordance with previous literature (*Adv. Mater.* **30**, 1707522 (2018). DOI: 10.1002/adma.201707522).

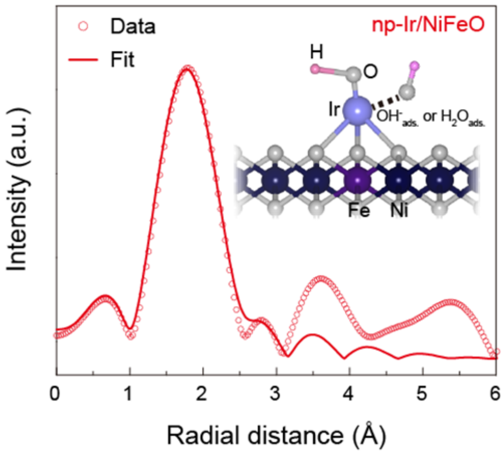

Figure R4 FT-EXAFS curve-fitting analysis

First-shell (Ir-O) fitting of FT-EXAFS spectrum for np-Ir/NiFeO. Inset shows the structure of optimized np-Ir/NiFeO.

NiOOH is one of the most active materials for the oxygen evolution reaction in alkaline media, which makes it difficult to understand the results in the manuscript. Unless authors provide conclusive evidence that iridium are the only oxygen-evolving active sites, it is not clear whether the Fe sites are also active (See for instance JACS 137 (2015) 1305. See also ACS Catal. 5 (2015) 5380).

Reply: Thank you for the insightful comment. We agree with the reviewer's point that Ni and Fe also are the active sites for oxygen evolution reaction in alkaline media. In our *operando* XAS analysis part, we not only proved that Ir sites are the efficient active sites for oxygen evolution reaction (**Fig. R5a-c**), but also testified the formation of Ni(Fe)OOH under realistic OER conditions. As shown in **Fig. R5d, e**, the absorption-edge of np-Ir/NiFeO displays an obvious positive shift during

electrochemical OER, indicating the transformation from hydroxides to oxyhydroxides in np-Ir/NiFeO. On the one hand, the formation of oxyhydroxides can further stabilize the isolated Ir atoms. On the other hand, we also consider that oxyhydroxides is one of the most active materials for OER in alkaline media. Therefore, we performed controlled *operando* XAS measurement of np-Ir/NiFeO and np-NiFeO to probe the effect of Ir doping on np-NiFeO (**Page 12, line 18-22** in revised manuscript and **Supplementary Fig. 24** in the revised Supplementary Information). It is observed that the incorporation of Ir atoms promotes the formation of Ni (Fe) oxyhydroxides, as evidenced by the greater shifts of the absorption-edge of np-Ir/NiFeO compared with that of np-NiFeO under realistic OER conditions. This is consistent with previous literature (*Adv. Mater.* **31**, 1901977 (2019). DOI: 10.1002/adma.201901977). Consequently, the increase of the number of active sites (including Ir, Ni, and Fe sites) is one of the reasons for the excellent OER performance of np-Ir/NiFeO.

Besides, we also considered the effect of Ir doping on Ni(Fe)OOH by DFT calculations (**Fig. 4a-c** in the revised manuscript). It is clear that the introduction of Ir could change the surface charge distribution of Ni and Fe sites, thus further improving the catalytic performance (*J. Am. Chem. Soc.* **140**, 3876-3879 (2018). DOI: 10.1021/jacs.8b00752). In the elementary reaction steps for the OER process in alkaline environments, the Gibbs free energy of the rate determining step for Ni sites and Fe sites dramatically decreased after the introduction of Ir atoms, suggesting a more favorable OER kinetics for Ni and Fe sites (please see next comment). Thus, the activation of the intrinsic activity of Ni (Fe) oxyhydroxides is another reason for the excellent OER performance of np-Ir/NiFeO.

In this work, we think both single-atom Ir and Ni(Fe)OOH support are active sites for OER. However, considering the high catalytic performance of single-atom Ir, we just focus on the dynamic behavior of single-atom Ir and try to find out the structure-property relationships based on Ir atoms, thus affording the rational design strategy of single-atom catalysts for OER at the atomic scale (**Fig. R5f**). In order to avoid the misunderstanding of readers, we have added relevant discussions (conclusion part) and literatures in the revised manuscript as follows:

Besides, the incorporation of Ir atoms could promote the formation of Ni (Fe) oxyhydroxides and arouse the intrinsic activity of Ni (Fe) oxyhydroxides, further accelerating the reactions.

Ref 14: Friebel, D. et al. Identification of highly active Fe sites in (Ni,Fe)OOH for electrocatalytic Water Splitting. *J. Am. Chem. Soc.* **137**, 1305-1313 (2015).

Ref 12: Diaz-Morales, O. et al. Guidelines for the rational design of Ni-based double hydroxide electrocatalysts for the oxygen evolution reaction. *ACS Catal.* **5**, 5380-5387 (2015).

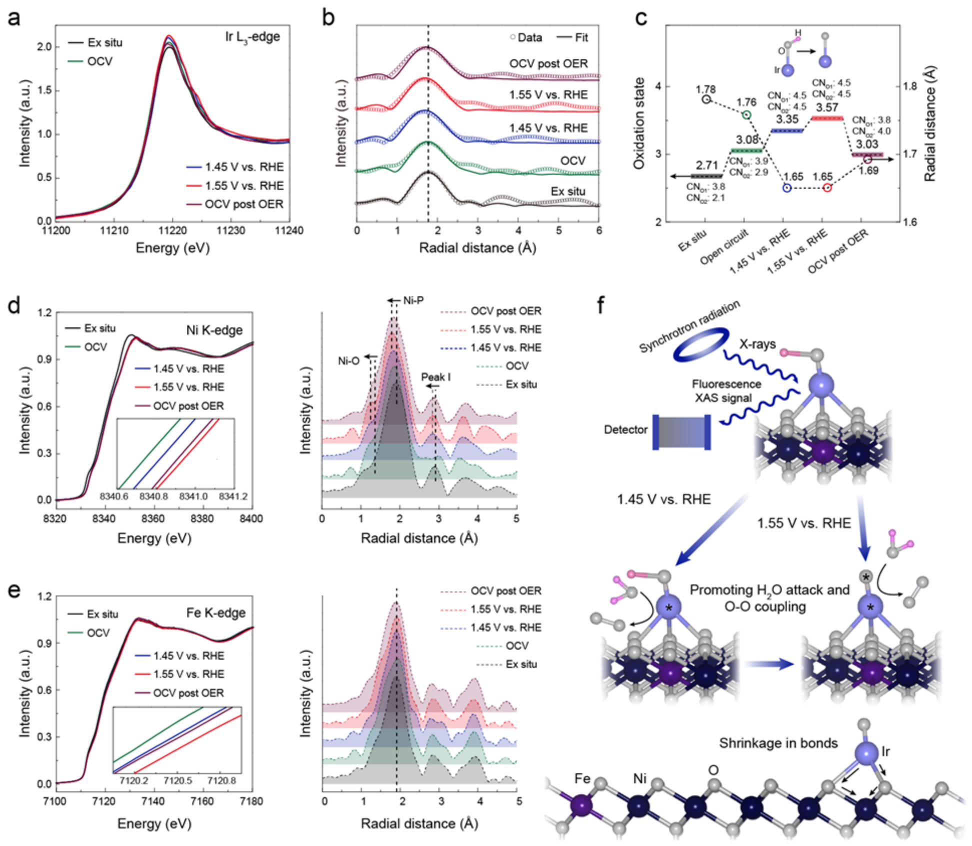

Figure R5 Operando XAS characterizations

(a) Operando XANES spectra of np-Ir/NiFeO recorded at Ir L_3-edge under different applied voltages from OCV to 1.55 V versus RHE in 1.0 M KOH. (b) Corresponding first-shell (Ir-O) fitting of FT-EXAFS spectra for np-Ir/NiFeO. (c) The fitted oxidation states from the white line intensity analysis, the variation of Ir-O bond, and the FT-EXAFS curve-fitting analysis. (d) Operando XANES spectra of np-Ir/NiFeO recorded at Ni K-edge under different applied voltages from OCV to 1.55 V versus RHE in 1.0 M KOH, as well as the corresponding FT-EXAFS spectra. (e) Operando XANES spectra of np-Ir/NiFeO recorded at Fe K-edge under different applied voltages from OCV to 1.55 V versus RHE in 1.0 M KOH, as well as the corresponding FT-EXAFS spectra. (f) Schematic illustration of the OER mechanism determined by the operando XAS analysis of np-Ir/NiFeO.

The reaction pathway (Fig 4b) was presented to support the hypothesis that iridium is the active site, based on the lower energy barrier to produce oxygen, but this figure is a bit confusing too. It is not clear to me, what Gibbs free energy is plotted in the y-axis of Fig 4b. One would expect 4.92 eV for the theoretical Gibbs free energy needed to transfer the four electrons during the oxygen evolution reaction (see J. Electroanal. Chem. 660 (2011) 254. See also ChemCatChem 3 (2011) 1159), although the energies depicted in Fig 4b are below 1.65 eV, which is thermodynamically not

possible for OER. Authors should elaborate further in the discussion of their computational DFT results.

Reply: We appreciate the reviewer's comment and suggestion. After reading these literatures carefully, we have revised the relevant calculations as follows:

The Gibbs free energy of species is plotted in the y-axis of **Fig. R6**. The free energy of the final species is 4.92 eV, which is also the free energy of the overall reaction.

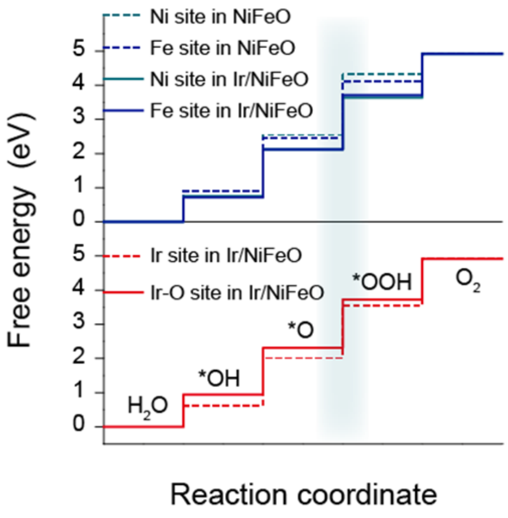

Figure R6 DFT calculations

Calculated free energy diagram of the OER. The blue box step is the rate determining step.

Besides, we have cited these relevant literatures in the revised manuscript to further support our calculations:

Ref 49: Koper, M. T. M. Thermodynamic theory of multi-electron transfer reactions: Implications for electrocatalysis. *J. Electroanal. Chem.* **660**, 254-260 (2011).

Ref 50: Man, I. C. et al. Universality in Oxygen Evolution Electrocatalysis on Oxide Surfaces. *ChemCatChem*, **3**, 1159-1165 (2011).

In this manuscript, authors propose that the enhanced nucleophilic attach of hydroxide anions over the oxo intermediate bound to iridium will lead to the O-O coupling and this explains the enhanced oxygen evolution reaction activity of the iridium-doped NiFeOOH. However, nucleophilic attack over the oxo intermediate is unlikely because its rich electron density (the Coulomb repulsion will make this attack very unlikely). The most accepted pathway toward for O-O coupling is reaction between the oxo intermediate and water from the aqueous electrolyte (see for instance J. Electroanal Chem 607 (2007) 83. See also ChemCatChem 3 (2011) 1159).

Reply: We appreciate the Reviewer's helpful suggestions. We feel sorry for

neglecting the influence of Coulomb repulsion between the oxo intermediate and hydroxide anions. According to the Reviewer's suggestions and relevant literature, we have revised the calculations as follows (**Fig. 4b**, **c** and **Method** in the revised manuscript. **Supplementary Note 3** in the revised Supplementary Information):

In this work, the scheme developed by Nørskov *et al.* was employed to gain an insight into the thermochemistry mechanism of OER elementary steps [21, 22]. The OER is assumed to involve four elementary reaction steps:
Step 1: $2H_2O + * \rightarrow *OH + H_2O + H^+ + e^-$
Step 2: $*OH + H_2O + H^+ + e^- \rightarrow *O + H_2O + 2(H^+ + e^-)$
Step 3: $*O + H_2O + 2(H^+ + e^-) \rightarrow *OOH + 3(H^+ + e^-)$
Step 4: $*OOH + 3(H^+ + e^-) \rightarrow *OO + 4(H^+ + e^-)$
where * represents the active site in the specific model.

Besides, we have cited these literatures in the revised manuscript and the revised Supplementary Information. We again thank the Reviewer for pointing out our defects in calculations.

Ref. 51 in the revised manuscript: Rossmeisl, J. et al. Electrolysis of water on oxide surfaces. *J. Electroanal. Chem.* **607**, 83-89 (2007).

Ref. 50 in the revised manuscript: Man, I. C. et al. Universality in Oxygen Evolution Electrocatalysis on Oxide Surfaces. *ChemCatChem*, **3**, 1159-1165 (2011).

Ref. 21 in the revised Supplementary Information: Rossmeisl, J. et al. Electrolysis of water on oxide surfaces. *J. Electroanal. Chem.* **607**, 83-89 (2007).

Ref. 22 in the revised Supplementary Information: Man, I. C. et al. Universality in Oxygen Evolution Electrocatalysis on Oxide Surfaces. *ChemCatChem*, **3**, 1159-1165 (2011).

Specific comments:
1. In the manuscript's introduction, authors states that "A state-of-the-art-catalyst with low overpotential and long with low overpotential and long-term stability is still challenging for industrial applications". This is true for acid-based oxygen evolution but not for the alkaline-based (i.e. the relevant case to the manuscript). In fact, there are commercially available (alkaline-based) electrolysers, operating with non-precious metals and having acceptably good performance. I would recommend to authors to rephrase this statement to be relevant to alkaline-based oxygen evolution.

Reply: We apologize for our inappropriate description. Following the comments and suggestions, we have rephrased this statement in the revised manuscript (**Page 2, line 17-18**):

However, their catalytic performance remains far from satisfactory.

2. In the discussion of the electrochemical performance (p. 8), it is indicated that the current density up to 300 mA / cm^2 could be due to large specific surface area. It is important for authors to discriminate between geometrical effects (i.e. surface roughness) and true catalytic effects. Authors indicate that the catalysts with single iridium atoms anchored depicted ca. 1.5-fold larger pseudo-capacitance than the undoped material, so surface area normalized activity should be presented to show that the Ir doping has a true catalytic effect.

Reply: Thank you for these comments and suggestions. According your suggestion, surface area normalized activity for np-Ir/NiFeO and np-NiFeO was calculated (**Note 1** and **Fig. R7**). Relevant figures and discussions have been added in the revised manuscript (**Page 9, line 14-19**) and the revised Supplementary Information (**Supplementary Note 2** and **Supplementary Fig. 19**):

Note 1: Calculation of the electrochemically active surface areas (ECSA).

The real surface area for OER is calculated from the ECSA, which can be converted from the specific capacitance. The specific capacitance for a flat surface is 40 μF cm^{-2} per cm^2_{ECSA}.

$$A_{ECSA}^{np-NiFeO} = \frac{14850\ \text{mF cm}^{-2}}{40\ \mu\text{F cm}_{geo}^{-2}\ \text{per cm}_{ECSA}^{2}} = 371.25\ \text{cm}_{ECSA}^{2}$$

$$A_{ECSA}^{np-Ir/NiFeO} = \frac{21350\ \text{mF cm}^{-2}}{40\ \mu\text{F cm}_{geo}^{-2}\ \text{per cm}_{ECSA}^{2}} = 533.75\ \text{cm}_{ECSA}^{2}$$

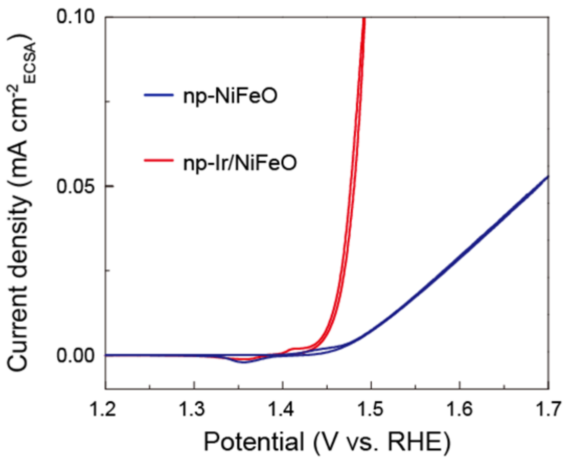

Figure R7. Polarization curves

Polarization curves of different samples normalized by the electrochemical active surface area (ECSA).

The ECSA-normalized CV curves are employed to emphasize the intrinsic activity. It is clear that the ECSA-normalized current density of np-Ir/NiFeO is larger than that of np-Ir/NiFeO, indicating that the higher OER activity of np-Ir/NiFeO results from not only the increased ECSA but also the enhanced intrinsic activity.

3. There is a typo in p. 8: hydrogen bubbles cannot be present at 1.48 V vs. RHE.

Reply: Thank you for pointing out this mistake. We have corrected this typo in the revised manuscript (**Page 8, line 15**).

4. To estimate the mass-normalized oxygen activity, authors are assuming that iridium is the only oxygen evolving sites, which is not totally clear (see the above discussion). To report this, authors should show that NiFe is not electrocatalytically active.

Reply: We appreciate you for this valuable suggestion. In the calculations of mass-normalized oxygen activity, the contribution of np-NiFeO has been deducted (**Supplementary Note 1** in the revised Supplementary Information). However, considering the regulation effect of Ir atoms incorporation, it is hard to get the accurate mass-normalized oxygen activity of Ir. Therefore, we have added further discussions to clarify the calculations of mass-normalized oxygen activity (**Supplementary Note 1** in the revised Supplementary Information):

Note that as the incorporation of Ir atoms could improve the catalytic activity of np-NiFeO support (including the intrinsic activity and the number of active sites), in the above calculations, the mass-normalized oxygen activity of Ir is the maximum value, and the actual mass-normalized oxygen activity of Ir is lower than this value.

Reviewers' Comments

Reviewer #1:
The revisions made are very satisfactory and the computational part of the manuscript is now very convincing. Given that the experimental parts are also deemed to be up to the standard of the journal by the other reviewers, I believe that the paper should be of high interest to the readers and can be published in Nature Communications.

Reviewer #3:
In the rebuttal of the manuscript entitled "Dynamic active-site generation of single-atomic iridium stabilized on nanoporous metal phosphides for accelerating water oxidation", Prof. Tan and co-workers have clarified the role of the single atomic iridium over the OER activity. The ECSA-normalized activity plot in the supplementary information significantly strengthened the statements about the enhancing effect of iridium sites on the OER activity. The revision on the computational part of the manuscript has also improved the clarity of these results and made it easier to link them with the experimental results and with the manuscript's statements and conclusions on the enhancing effect of iridium. However, I encourage the authors to further elaborate on their hypothesis about the role of iridium sites in promoting the formation of the nickel-iron oxyhydroxide (i.e. nickel/iron oxidation) because I think this is a critical to understand the OER activity enhancement reported

in the manuscript. I suggest the authors to check the reported observations on OER activity enhancement of MnOx supported in different noble metals (see for instance J. Phys. Chem. Lett. 6 (2015) 4178. See also ACS Sustainable Chem. Eng. 3 (2015) 2049).
After further elaborating on the role of Ir on the formation of NiFeOxHy, I would recommend the publication of this manuscript in Nature Communication.

Response to Reviewers' Comments

Reviewer #1:
The revisions made are very satisfactory and the computational part of the manuscript is now very convincing. Given that the experimental parts are also deemed to be up to the standard of the journal by the other reviewers, I believe that the paper should be of high interest to the readers and can be published in Nature Communications.

Reply: We appreciate your recommendation of acceptance and helpful comments in the reviewing process and are pleased to have our manuscript be reviewed by you.

Reviewer #3:
In the rebuttal of the manuscript entitled "Dynamic active-site generation of single-atomic iridium stabilized on nanoporous metal phosphides for accelerating water oxidation", Prof. Tan and co-workers have clarified the role of the single atomic iridium over the OER activity. The ECSA-normalized activity plot in the supplementary information significantly strengthened the statements about the enhancing effect of iridium sites on the OER activity. The revision on the computational part of the manuscript has also improved the clarity of these results and made it easier to link them with the experimental results and with the manuscript's statements and conclusions on the enhancing effect of iridium. However, I encourage the authors to further elaborate on their hypothesis about the role of iridium sites in promoting the formation of the nickel-iron oxyhydroxide (i.e. nickel/iron oxidation) because I think this is a critical to understand the OER activity enhancement reported in the manuscript. I suggest the authors to check the reported observations on OER activity enhancement of MnOx supported in different noble metals (see for instance J. Phys. Chem. Lett. 6 (2015) 4178. See also ACS Sustainable Chem. Eng. 3 (2015) 2049).
After further elaborating on the role of Ir on the formation of NiFeOxHy, I would recommend the publication of this manuscript in Nature Communication.

Reply: We are very grateful to your encouraging and positive comments. As you suggested, we add further description with the following sentence:
Besides, further insights into the regulation effect of Ir atoms incorporation by controlled operando XAS measurement of np-Ir/NiFeO and np-NiFeO confirm that more transformation from Ni (Fe) oxides or hydroxides to Ni (Fe) oxyhydroxides

occurs on the surface of np-Ir/NiFeO than np-NiFeO, as evidenced by the greater shifts of the absorption-edge of np-Ir/NiFeO compared with that of np-NiFeO under OER conditions. Considering the existence of strong metal-support interactions in SACs, it is reasonable to believe that the charge transfer from the Ni (Fe) oxides or hydroxides to Ir atoms at the interfacial sites under realistic OER conditions is responsible for the facilitating of oxidation of Ni (Fe) oxides or hydroxides to Ni (Fe) oxyhydroxides, thus enabling an earlier onset of current for the OER [49, 50].

Besides, we have cited these relevant literatures in the revised manuscript to further support this conclusion:

Ref. 49: Seitz, L. C. et al. Enhancement Effect of Noble Metals on Manganese Oxide for the Oxygen Evolution Reaction. *J. Phys. Chem. Lett.* **6**, 4178-4183 (2015).

Ref. 50: Zhang, H. et al. Enhanced Interactions between Gold and MnO_2 Nanowires for Water Oxidation: A Comparison of Different Chemical and Physical Preparation Methods. *ACS Sustainable Chem. Eng.* **3**, 2049-2057 (2015).
